# Transforming protein-polymer conjugate purification by tuning protein solubility

**DOI:** 10.1038/s41467-019-12612-9

**Published:** 2019-10-17

**Authors:** Stefanie L. Baker, Aravinda Munasinghe, Bibifatima Kaupbayeva, Nin Rebecca Kang, Marie Certiat, Hironobu Murata, Krzysztof Matyjaszewski, Ping Lin, Coray M. Colina, Alan J. Russell

**Affiliations:** 1 Department of Biomedical Engineering, Scott Hall 4N201, Carnegie Mellon University, Pittsburgh, PA 15213 USA; 2 Center for Polymer-Based Protein Engineering, Carnegie Mellon University, Pittsburgh, PA 15213 USA; 3 Department of Chemistry, 354 Leigh Hall, University of Florida, Gainesville, FL 32611 USA; 4 George and Josephine Butler Polymer Research Laboratory, University of Florida, Gainesville, FL 32611 USA; 5 Center for Macromolecular Science and Engineering, University of Florida, Gainesville, FL 32611 USA; 6 Department of Biological Sciences, Carnegie Mellon University, Pittsburgh, PA 15213 USA; 7 Department of Chemical Engineering, Carnegie Mellon University, Pittsburgh, PA 15213 USA; 8 Université Paul Sabatier, Toulouse, 31062 France; 9 Department of Chemistry, Carnegie Mellon University, Pittsburgh, PA 15213 USA; 10 Department of Materials Science and Engineering, University of Florida, Gainesville, FL 32611 USA

**Keywords:** Biomaterials - proteins, Biocatalysis, Enzymes, Protein structure predictions

## Abstract

Almost all commercial proteins are purified using ammonium sulfate precipitation. Protein-polymer conjugates are synthesized from pure starting materials, and the struggle to separate conjugates from polymer, native protein, and from isomers has vexed scientists for decades. We have discovered that covalent polymer attachment has a transformational effect on protein solubility in salt solutions. Here, protein-polymer conjugates with a variety of polymers, grafting densities, and polymer lengths are generated using atom transfer radical polymerization. Charged polymers increase conjugate solubility in ammonium sulfate and completely prevent precipitation even at 100% saturation. Atomistic molecular dynamic simulations show the impact is driven by an anti-polyelectrolyte effect from zwitterionic polymers. Uncharged polymers exhibit polymer length-dependent decreased solubility. The differences in salting-out are then used to simply purify mixtures of conjugates and native proteins into single species. Increasing protein solubility in salt solutions through polymer conjugation could lead to many new applications of protein-polymer conjugates.

## Abstract (web summary)

Therapeutic proteins are often conjugated with polymers, but separating the conjugate from unconjugated protein and free polymer is a major challenge. Here, the authors discover that proteins conjugated to charged or zwitterionic polymers maintain solubility in 100% ammonium sulfate, greatly simplifying purification.

## Introduction

Purification of proteins in biotechnology, typically by chromatography, accounts for 30–50% of manufacturing costs of high-value biopharmaceuticals and is a major challenge that is often undervalued in this $150 billion dollar industry. Purification via precipitation has been historically used for fractionation of biomolecules from blood plasma, but is becoming increasingly prevalent for other therapeutic biologics. Indeed, a recent study found that from 2010 to 2017, the number of precipitation patents has steadily increased totaling 246 (ref. ^1^). Most notably, the number of patents specifically for antibody purification via precipitation has shown the largest growth due to the ever-expanding fraction of antibodies being used therapeutically (both unmodified and PEGylated (PEG: polyethylene glycol) forms)^1^. As such, large pharmaceutical companies, including Amgen and Novartis, have increased their research and development investments to support this expansion^1^. There is an increasing demand for more efficient, simple, cost effective, and scalable protein purification methods, and precipitation methods have started to gain the most attention to help meet manufacturing needs.

Covalently attaching synthetic polymers to a protein, such as PEGylation, is one way to alter the bioactivity^2^, stability^3^, circulating half-life^4^, and immunogenicity^5^ of the resultant protein–polymer conjugate. Polymer attachment site^6^, number of attachments^7,8^, polymer type^3,9^, polymer chain length^10,11^, and conjugation chemistry^12,13^ are all variables that can be tuned to optimize the conjugate’s properties for a specific outcome, such as increased thermostability^14^ or solubility. Protein solubility is especially important for therapeutic proteins, which can require concentrations as high as 100 mg/mL for effective dose administration. High concentrations of proteins unfortunately enhance aggregation-based degradation^15^. Protein aggregation caused by poor solubility has also been linked to various disease states. For example, neurological diseases, such as Parkinson’s or Alzheimer’s, are caused by protein aggregation. Additionally, γD-crystallin’s P23T (Pro23 to Thr) mutation reduces solubility and causes early onset of cataracts^16^. In industrial biotechnology, enzyme solubility in non-aqueous media is also of particular interest because new catalytic reactions become possible and more efficient due to increased solubilities of nonpolar substrates/products and reduction of unwanted hydrolytic side-reactions^17–21^.

Protein solubility depends on numerous intrinsic and extrinsic factors. The intrinsic chemical structure of the protein surface and the number of charged amino acids influence solubility^22^. In aqueous solutions, solubility is proportional to the number of charged amino acids on the protein surface^22^. Interestingly, proteins are least soluble at their isoelectric point (pI) where they have no net charge^22^. Therefore, chemical modification of the protein surface can alter solubility. Indeed, PEGylation of proteins and other hydrophobic drugs increases their solubility in water^23^, while conjugation of poly(2-(dimethylamino)ethyl methacrylate (pDMAEMA) was found to facilitate the molecular dissolution of α-chymotrypsin in acetonitrile^24^. Recently, pH-responsive polymers were conjugated to Protein-A to aid in the controlled precipitation of antibodies from cell culture supernatants^25^. Extrinsic factors, including temperature, pH, ionic strength, and other additives can also impact solubility. It is difficult to accurately determine intrinsic protein solubility because many proteins are highly soluble requiring large amounts of lyophilized protein to reach saturation in a given volume. For this reason, additives, such as salts, long-chain polymers, or organic solvents, are often used to precipitate proteins to determine solubility^26–28^. Controlling protein solubility is at the very core of the biotechnology industry, since protein precipitation is an essential first step in almost all protein purification protocols.

The ability of a salt to precipitate a protein can be predicted by the Hofmeister series where kosmotropic salts stabilize protein structure and induce salting-out, while chaotropic salts destabilize and promote salting-in^28^. Ammonium sulfate ((NH_4_^+^)_2_SO_4_^2−^) is strongly kosmotropic and has one of the highest solubilities in water (4.1 M at 25 °C) making it one of the most effective salts for protein precipitation without causing denaturation. In solution, proteins are always surrounded by a layer of water molecules known as the hydration layer. These water molecules interact with the protein surface through hydrogen bonding and electrostatic interactions and are essential for maintaining protein structure, dynamics, and bioactivity^29^. As the salt concentration is increased, the water molecules become attracted to the salt ions and are pulled away from the protein’s surface. Eventually, the hydration layer is depleted which promotes protein–protein hydrophobic interactions and after enough aggregation, the proteins precipitate. The salting-out point is different for each protein since each protein has a different surface charge composition and solubility. Ammonium sulfate precipitation is the principal technique in biotechnology used for both purification of a protein of interest from a crude mixture and for concentrating dilute solutions^30,31^.

Since proteins precipitate at a certain salt concentration, most organisms cannot survive in high salinity due to cytoplasmic protein aggregation. However, halophiles have adapted to living in areas containing high salts, such as the Dead Sea or the Great Salt Lake^32,33^. There are two mechanisms for how halophiles are able to do this. Halophiles accumulate osmolytes, such as betaines, in their cytoplasm that help control osmotic pressure while stabilizing proteins^34,35^. Halophiles have also evolved to control cellular salt fluxes and they have specially adapted intracellular proteins that withstand high salt concentrations^32^. Halophilic proteins have an abundance of negatively charged amino acids (aspartic acid and glutamic acid), short polar side chains, increased hydrophilicity, lower helical formation, and higher coil formation^33,36^. Halophiles have also been categorized depending on the NaCl concentration they survive in where slight halophiles thrive in 0.34–0.85 M salt, moderate halophiles in 0.85–3.4 M salt, and extreme halophiles in 3.4–5.1 M salt^37^.

The intracellular betaine osmolytes used by some halophiles mimic the structure of zwitterionic polymer side chains originating from monomers, such as 3-[[2-(methacryloyloxy) ethyl]dimethylammonio]propionate. Indeed, many studies have been performed to determine the behavior of zwitterionic polymers in salt solutions for applications in antifouling, antibacterial surfaces, surface wetting, and anti-icing^38–40^. We hypothesized that modifying the surface of a protein with rationally designed polymers would predictably tune protein solubility at high salt concentrations. In this paper, we use protein-initiated grafted-from atom transfer radical polymerization (ATRP) to create large families of protein–polymer conjugates with desired salting-out behaviors. We vary the number of polymer chains (grafting density), polymer length (degree of polymerization, DP), and polymer type on a model protein, lysozyme (Lyz). Protein–polymer conjugate salting-out points are determined by ammonium sulfate precipitation. We also determine changes in hydrodynamic diameters and stabilities over time in increasing ammonium sulfate concentrations using dynamic light scattering. Furthermore, we measure the enzymatic activities of the conjugates in 4.1 M ammonium sulfate. Atomistic molecular dynamic simulations provide mechanistic explanations for changes in conjugate solubility. Finally, we utilize the difference in salting-out points of native protein and protein–polymer conjugates to purify the conjugates from a heterogeneous mixture.

## Results

### Conjugate synthesis and characterization

**Fig. 1:**
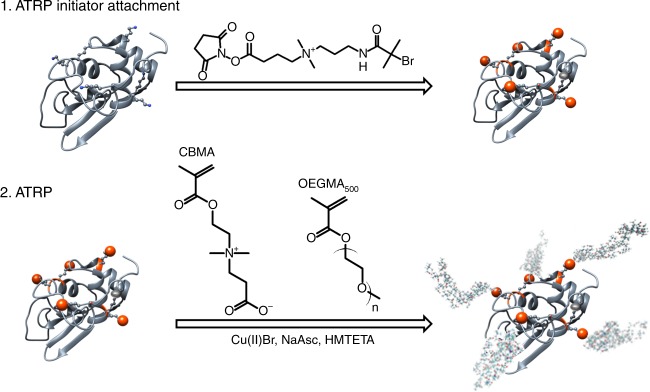
Grafting-from Lyz–polymer conjugate synthesis using ATRP. A positively charged ATRP initiator was first reacted with accessible amino groups on Lyz’s surface. Next, ATRP was used to grow polymers of zwitterionic CBMA or neutral OEGMA at increasing polymer lengths. Additional acronyms: sodium ascorbate (NaAsc), 1,1,4,7,10,10-Hexamethyltriethylenetetramine (HMTETA)

Lysozyme–polymer conjugates were synthesized with a high grafting density and varied polymer chain lengths using grafting-from ATRP (Fig. 1). Five different chain lengths of two different polymer types were chosen to study the effect of polymer attachment on solubility: zwitterionic poly(carboxybetaine methacrylate) (pCBMA) and neutral poly(oligo(ethylene glycol) methacrylate) (pOEGMA). These polymers also have significantly different octanol-water distribution coefficients (logD) where the logD of CBMA monomer is ~−2.35 and OEGMA is ~0.84 (ref. ^3^). Clearly, CBMA is more hydrophilic than OEGMA and while both are net neutral, CBMA is highly charged. Small molecule positively charged ATRP initiators^13^ were first reacted with the available seven amino groups on Lyz’s surface. The number of reacted initiators was determined by the change in mass of Lyz-initiator compared to native Lyz analyzed by matrix-assisted laser desorption/ionization time of flight (MALDI-ToF) mass spectrometry (Supplementary Fig. 1). The average of 5 attached initiators (5+) were the sources for polymer growth via ATRP. Polymer chain length was increased by increasing the monomer to initiator ratio in the ATRP reaction (targeted DPs from 25 to 200). Lyz–polymer conjugates were purified via dialysis and were then lyophilized.

A bicinchoninic acid (BCA) protein assay was used to determine protein concentration in the conjugate samples from which polymer concentration, molecular mass, and degree of polymerization were estimated^41^. Targeted DPs during ATRP were 25, 50, 100, 150, and 200, yielding measured DPs of 18, 32, 56, 79, and 91 for pCBMA conjugates and 25, 43, 90, 105, 164 for pOEGMA conjugates, respectively (Table 1).

**Table 1:** Lyz–polymer characterization

Sample^a^	Estimated DP^a^	*D*_h_ (nm)	Cleaved polymer *M*_n_ (kDa)	*Đ*
Lyz	–	3.6 ± 0.1	–	–
Lyz(5+)pCBMA_25_	18	7.9 ± 0.4	8.1	1.4
Lyz(5+)pCBMA_50_	32	11.0 ± 1.0	11.9	1.6
Lyz(5+)pCBMA_100_	56	13.4 ± 0.9	20.9	1.7
Lyz(5+)pCBMA_150_	79	15.4 ± 0.8	30.8	1.8
Lyz(5+)pCBMA_200_	91	16.8 ± 0.8	38.7	1.9
Lyz(5+)pOEGMA_25_	25	9.2 ± 0.8	17.5	1.7
Lyz(5+)pOEGMA_50_	43	12.6 ± 1.1	26.9	1.8
Lyz(5+)pOEGMA_100_	90	20.2 ± 1.7	46.6	1.9
Lyz(5+)pOEGMA_150_	105	22.2 ± 2.9	53.2	1.8
Lyz(5+)pOEGMA_200_	164	26.2 ± 3.0	85.4	1.7

Data was collected using a bicinchoninic acid (BCA) assay to estimate degree of polymerization (DP), dynamic light scattering number distribution to measure hydrodynamic diameter (*D*_h_), and acid hydrolysis with gel permeation chromatography (GPC) to calculate number-average molecular mass (*M*_n_) and dispersity (*Đ*) of cleaved polymer. DLS data are presented as mean number distributions ± 1 standard deviation error bars

^a^Subscript numbers represent the targeted DP from the ATRP reaction. Estimated DPs are calculated from the BCA assay^41^. The (5+) represents the number of positively charged initiators on the conjugate

Conjugates were next characterized by dynamic light scattering (mean number distribution ± 1 standard deviation error bar) in phosphate buffered saline (PBS) to determine how an increase in chain length correlated with increased conjugate size (Supplementary Fig. 2). Native Lyz had a hydrodynamic diameter of 3.6 ± 0.1 nm, Lyz-pCBMA conjugates increased in hydrodynamic diameters from 7.9 ± 0.4 nm (DP 18) to 16.8 ± 0.8 nm (DP 91), and Lyz-pOEGMA conjugates increased in hydrodynamic diameters from 9.2 ± 0.8 nm (DP 25) to 26.2 ± 3.0 nm (DP 164) (Table 1). Additionally, polymers were cleaved from Lyz by acid hydrolysis and were analyzed by gel permeation chromatography (GPC) for molecular mass (*M*_n_) and dispersity (*Đ*). Polymer *M*_n_ increased from 8.1 kDa (*Đ* 1.4) to 38.7 kDa (*Đ* 1.9) for pCBMA and from 17.5 kDa (*Đ* 1.7) to 85.4 kDa (*Đ* 1.7) for pOEGMA (Table 1 and Supplementary Figs. 3 and 4).

### Effect of polymer length on conjugate solubility

**Fig. 2:**
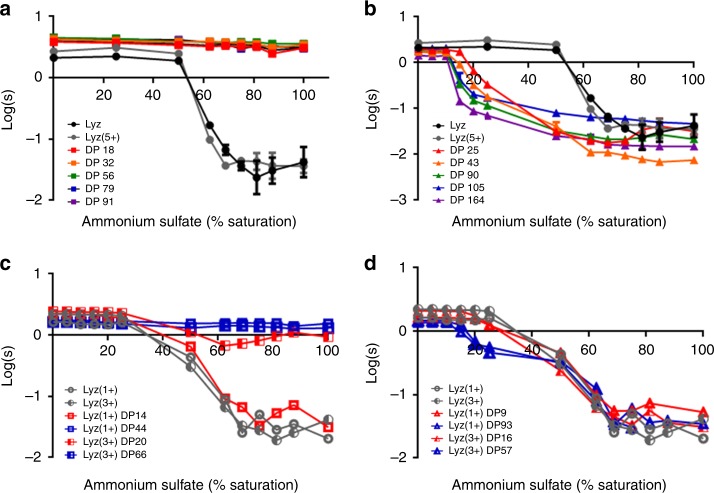
Ammonium sulfate precipitation of conjugates. **a**, **b** Native Lyz, Lyz(5+), and Lyz–polymer conjugates. Plots are solubility (log of the supernatant protein concentration) versus ammonium sulfate percent saturation. 100% saturation corresponds to 4.1 M salt concentration. **a** Lyz(5+)pCBMA conjugates with DP 18, DP 32, DP 56, DP 79, and DP 91. **b** Lyz(5+)pOEGMA conjugates with DP 25, DP 43, DP 90, DP 105, and DP 164. pCBMA increased Lyz’s solubility while pOEGMA decreased Lyz’s solubility depending on DP. **c**, **d** Ammonium sulfate precipitation of native Lyz(1+), Lyz(3+), and Lyz–polymer conjugates with lower grafting densities and low/high DP. **c** pCBMA conjugates of Lyz(1+) DP 14, Lyz(1+) DP 44, Lyz(3+) DP 20, and Lyz(3+) DP 66. The only pCBMA conjugate that precipitated was the lowest grafting density and lowest DP. **d** pOEGMA conjugates of Lyz(1+) DP 9, Lyz(1+) DP 93, Lyz(3+) DP 16, and Lyz(3+) DP 57. pOEGMA length affected solubility more than grafting density. Error bars represent the standard deviations from triplicate measurements (*n* = 3)

Native Lyz, Lyz-initiator, and Lyz–polymer conjugates were subjected to precipitation by ammonium sulfate at pH 7.0 to determine their salting-out points (Fig. 2). Lyz has been shown to salt-out as predicted by the anion Hofmeister series at basic pH values and high ionic strength, but salt-out according to the reversed anion Hofmeister series at neutral to acidic pH and moderate ionic strength^42,43^. Additionally, Lyz solubility can be predicted from the cation Hofmeister series when pH < pI (Lyz pI: ~11)^44^. Native Lyz, as expected^22^, precipitated around 60% saturated ammonium sulfate (2.5 M) (Fig. 2a, b). Lyz-initiator also precipitated around 60% saturation. A charge-preserving ATRP initiator^13^ was used to synthesize the Lyz-conjugates so that the positive charges on amino groups were retained after initiator attachment. Therefore, the net numbers of positive and negative charges on the protein surface were preserved after initiator attachment causing Lyz-initiator to salt-out at a similar salt concentration to native Lyz.

We expected that increasing pCBMA length would increase conjugate hydrophilicity and should therefore increase the salt concentration needed to salt-out Lyz-pCBMA conjugates since free zwitterionic polymers are highly solvated in water and solvation increases with salt concentration^45,46^. Lyz(5+)pCBMA conjugates actually exhibited no salting-out behavior, independent of DP, even up to 100% saturation (4.1 M) (Fig. 2a). Very few native proteins are soluble in 100% ammonium sulfate (besides some halophilic proteins) and most precipitate in the range of 40–60% saturation. Saturated ammonium sulfate is, after all, ~seven times the ionic strength of seawater. Conversely, Lyz(5+)pOEGMA conjugates exhibited a length-dependent reduction in salting-out concentration (Fig. 2b). Although the net surface charge on Lyz-pOEGMA was similar to native Lyz, the dense molecular shell of uncharged, amphiphilic polymers undoubtedly increased the hydrophobicity of the entire complex. Increasing the pOEGMA chain length decreased the conjugate’s solubility. Long-chained Lyz(5+)pOEGMA with a DP of 164 precipitated around 10% saturation and the salting-out point increased according to DP where short-chained DP 25 Lyz(5+)pOEGMA did not precipitate until about 20% saturation. We surmised that conjugate hydrophobicity increased with pOEGMA length^47^.

We were interested in whether the observed differences in solubilities were due to covalent polymer conjugation versus the presence of polymer in solution since proteins have been precipitated non-covalently by some PEG’s^48,49^. A more recent study found that unattached (free) charged polymers with the size 25 times larger than the protein’s diameter could induce protein precipitation by wrapping themselves around the protein to neutralize surface charges^50^. We therefore synthesized free pCBMA and pOEGMA and performed ammonium sulfate precipitation of native Lyz in the presence of free polymers (Supplementary Figs. 5 and 6). The concentrations of free polymers used matched the mass concentrations of polymers in the long-chained Lyz(5+)pCBMA (DP 91) and Lyz(5+)pOEGMA (DP 164) ammonium sulfate precipitation samples. The presence of free polymers did not affect Lyz solubility and Lyz precipitated around 60% saturation in the presence of both pCBMA and pOEGMA (Supplementary Fig. 5), showing that covalent attachment of the polymer to the protein was required in order to tune the solubility of the conjugates in salt solutions.

### Effect of grafting density on conjugate solubility

Since all of the Lyz(5+)pCBMA conjugates remained soluble up to 100% saturation and Lyz(5+)pOEGMA conjugates precipitated at relatively low percent saturations, we next investigated whether there was a minimum amount of polymer that would elicit a change in the salting-out point from native Lyz. We next synthesized Lyz-pCBMA and Lyz-pOEGMA conjugates with lower grafting densities, namely 1 and 3 average initiator modifications (Supplementary Fig. 7). From each Lyz-initiator, short and long-chained pCBMA and pOEGMA were grown. Conjugates were characterized using a BCA assay to estimate DP^41^ and DLS to determine hydrodynamic diameter (Supplementary Table 1).Lyz with 1 initiator had pCBMA’s of DP 14 (5.2 ± 0.8 nm; mean ± SD) or DP 44 (6.0 ± 0.8 nm) and pOEGMA’s of DP 9 (5.9 ± 0.8 nm) or DP 93 (14.0 ± 2.5 nm). Lyz with 3 initiators had pCBMA’s of DP 20 (5.3 ± 1.1 nm) or DP 66 (12.7 ± 1.5 nm) and pOEGMA’s of DP 16 (7.9 ± 1.5 nm) or DP 57 (18.0 ± 3.3 nm).

Ammonium sulfate precipitation of the variable grafting density conjugates (Fig. 2c, d) showed that the conjugate with the least amount of polymer, Lyz(1+)pCBMA DP 14 did not remain soluble in saturated ammonium sulfate. For Lyz-pOEGMA, polymer length, rather than grafting density, influenced the salting-out point. Long chain pOEGMA conjugates with 1 or 3 initiators precipitated first (around 20% saturation) while short-chained pOEGMA conjugates precipitated similarly to Lyz-initiator (at around 60% saturation). The range of concentrations over which the pOEGMA conjugates precipitated could have been related to heterogeneity within the samples (not every conjugate molecule has the same number of polymer chains attached). Indeed, ammonium sulfate precipitation should be an excellent route to fractionating heterogeneous polymer-protein conjugate solutions.

### Zwitterionic conjugate stability in ammonium sulfate

**Fig. 3:**
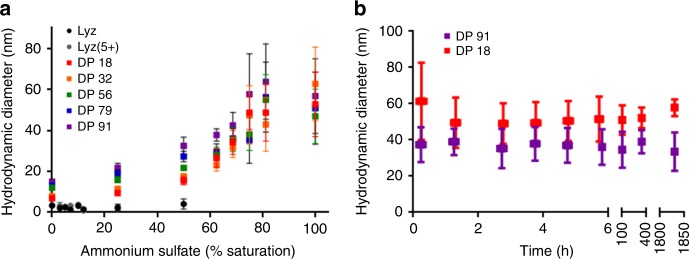
Dynamic light scattering data to measure hydrodynamic diameters. **a** Lyz(5+)pCBMA conjugates in increasing ammonium sulfate saturation for DP 18, DP 32, DP 56, DP 79, and DP 91. All conjugates increased in hydrodynamic diameter with increased ammonium sulfate concentration. Native Lyz and Lyz(5+) hydrodynamic diameters were not able to be measured after 50% saturation because samples precipitated. **b** Lyz(5+)pCBMA DP 18 and DP 91 hydrodynamic diameter stability over 2.5 months in 100% saturated ammonium sulfate. Data are presented as number distribution averages ± 1 standard deviation errors from *n* = 3 measurements)

Since all Lyz(5+)pCBMA conjugates had solubilities up to 100% saturated ammonium sulfate, we next investigated how their hydrodynamic diameters changed with increasing salt concentration and whether the size of the conjugates changed over time. Ammonium sulfate precipitation was performed again on Lyz(5+)pCBMA conjugates and DLS measurements of the supernatants were taken at each increasing ammonium sulfate concentration (Fig. 3a). The hydrodynamic diameters of native Lyz and Lyz-initiator were relatively stable up to 50% saturation. Beyond this point, the samples precipitated and DLS measurements of the supernatants were not able to be performed. Lyz(5+)pCBMA conjugates, however, displayed reversible (Supplementary Fig. 8) increases in hydrodynamic diameters up to 100% saturation where the hydrodynamic diameters of all conjugates were around 60 nm by number distribution. Additionally, the standard deviation in the measurements increased as salt concentration increased.

Next, we determined the polymer length effect on the rate of change in conjugate *D*_h_ during storage in saturated ammonium sulfate solution (Fig. 3b). The hydrodynamic diameters of both the short DP 18 and long DP 91 conjugates were stable for up to 2.5 months. Storage of protein solutions, especially for pharmaceuticals, in ammonium sulfate is of great interest because it inhibits bacterial growth and prevents contamination during shelf storage^51^.

The increases in pCBMA conjugate size with salt concentration could be attributed to either micro-aggregation or an actual change in conjugate size. Although single peaks were detected in number distributions up to 100% saturation, multimodal peaks became prominent in the volume distributions at 70% saturation (Supplementary Fig. 9). At approximately this salt concentration, we also noted the increase in the standard deviations of the DLS measurements. This was indicative of a small degree of micro-aggregation, which did not result in precipitation, at ammonium sulfate concentrations above 70%. An actual change in conjugate size could have resulted from the anti-polyelectrolyte effect. The anti-polyelectrolyte effect of zwitterionic polymers has been studied in depth for non-fouling biomaterials, but has not been studied for the polymers bound to proteins^40,45,52,53^. Zwitterionic polymers are composed of an equal number of positive and negative charges. Intra- and inter-chain electrostatic interactions cause the polymer to adopt a more collapsed conformation in water. As salt concentration is increased, the salt ions neutralize the electrostatic interactions and allow the polymer chains to extend in solution and become more hydrated. Although the data reported herein would be the first direct observation of this effect in a protein–polymer conjugate, the anti-polyelectrolyte chain extension could have contributed to the observed increase in hydrodynamic diameter. Changes in polymer conformation caused by anti-polyelectrolyte effects would also increase intrinsic viscosity^53,54^. DLS actually measures the diffusion coefficient of a particle in solution due to Brownian motion and converts this parameter to a hydrodynamic diameter using the Stokes-Einstein equation. Therefore, an increase in intrinsic viscosity would decrease the diffusion coefficient of a particle and increase hydrodynamic diameter^46,55^. We therefore sought to determine whether zwitterionic polymer chains might extend as a function of salt concentration, and how such behavior would differ from pOEGMA-protein conjugates at the atomistic level.

### Protein–polymer conjugate atomistic MD simulations

To determine the molecular basis for why zwitterionic polymers conjugated to proteins prevented salting-out, atomistic molecular dynamics (MD) simulations were performed. On one hand, the conjugated zwitterionic polymers could have prevented depletion of the hydration layer around the protein. Alternatively, the hydration layer could be depleted, but the shell of highly charged zwitterionic polymers prevented proteins from aggregating and precipitating. We additionally sought to determine the mechanism for the early precipitation of Lyz-pOEGMA from MD.

**Fig. 4:**
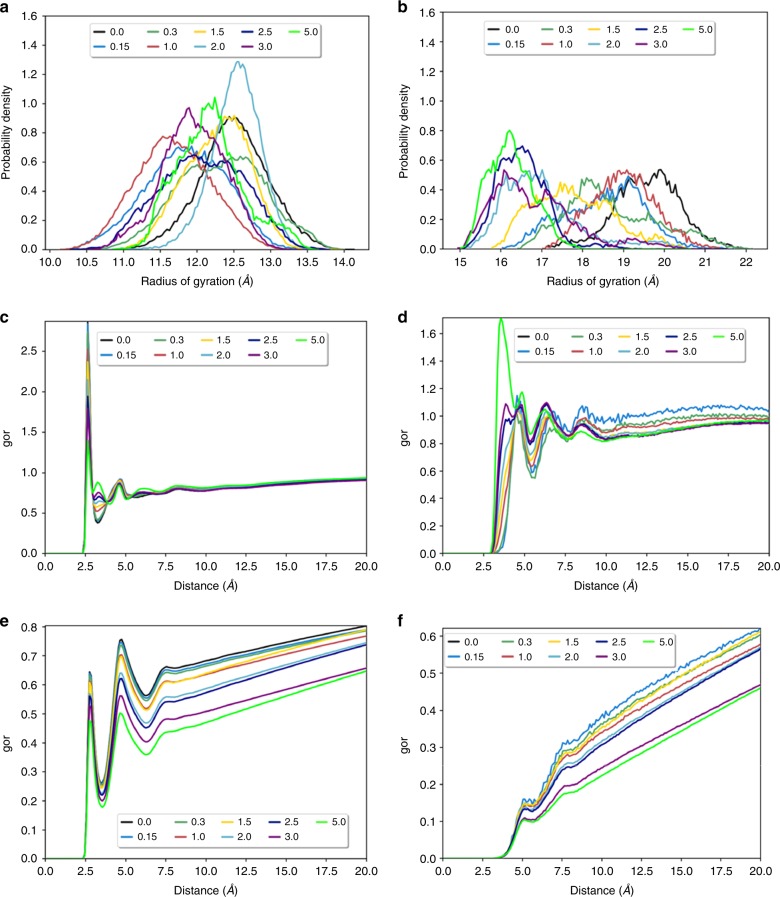
Conjugate size and hydration state from MD simulations. Radius of gyration of **a** free pCBMA and **b** free pOEGMA in increasing NaCl concentration. Radial distribution functions (RDF) for Lyz(5+)pCBMA DP 18 between **c** water molecules and O^−^ atoms of pCBMA and **d** Cl^−^ ions and O^−^ atoms of pCBMA in increasing NaCl concentrations. Hydration layer increases as NaCl concentration increases and both water molecules and Cl^−^ ions become more ordered. Radial distribution function analyses for Lyz(5+)pOEGMA DP 25 between **e** water molecules and PEG in the pOEGMA monomer side-chain and **f** Cl^−^ ions and PEG in the pOEGMA monomer side-chain in increasing NaCl. Hydration decreases with increasing salt for Lyz(5+)pOEGMA

Models of short-chained Lyz(5+)pCBMA (DP 18) and Lyz(5+)pOEGMA (DP 25) were built in silico attached to K1, K13, K33, K97, and K116, which were the lysine residues with the most exposed surface areas from a tertiary structure-based prediction^56^. Simulations were performed over 500 and 200 ns for Lyz-pCBMA and Lyz-pOEGMA, respectively, in increasing NaCl concentrations from 0.0 to 5.0 M. Simulations were also performed on free, unconjugated pCBMA chains (DP 18) and free pOEGMA chains (DP 25) (with positively charged initiators). First, the radii of gyrations (*R*_g_’s) of free polymers were determined in increasing NaCl concentrations to determine the effect of salt on polymer conformations. pCBMA’s average *R*_g_ was ~12 ± 1.5 Å and while the average fluctuated slightly with NaCl concentration, there was no observable correlation indicating that pCBMA polymer chains sampled similar conformations (Fig. 4a). Conversely, there was a significant change in the *R*_g_ of pOEGMA as a function of increasing salt concentration (Fig. 4b). At 0 M NaCl, pOEGMA’s average *R*_g_ was ~20 Å and the *R*_g_ steadily decreased to 16 Å at 5 M NaCl. This indicated that pOEGMA chains were collapsing with increasing salt concentration. These trends were consistent between the free polymer and conjugate simulations (Supplementary Fig. 10 and Supplementary Movie 1).

In order to determine the local arrangement of solvent molecules (water, Na^+^ and Cl^−^ ions) near the conjugates, radial distribution function (RDF) analyses were performed between the solvent molecules and the O^−^ atoms in pCBMA side-chains or PEG’s in the pOEGMA side-chains. Essentially, these calculations determined the probability of finding solvent molecules within a certain distance of the polymer chains. For Lyz(5+)pCBMA, as the NaCl concentration increased, an additional solvation layer emerged corresponding to the appearance of a peak around 3.5 Å while maintaining the additional peaks at distances up to ~10.0 Å (Fig. 4c). Furthermore, the dominant peak around 2.5 Å decreased in intensity as NaCl concentration increased indicating that some of the water molecules that were previously in the 1st hydration layer moved further away to form the additional hydration layers. Moreover, as salt concentration increased, the presence of nearby Cl^−^ ions increased and Na^+^ ions decreased. This can be effectively seen by the increase in the peak intensity around 3.5 Å at 5.0 M NaCl (Fig. 4d and Supplementary Fig. 11). This phenomenon aligns well with the anti-polyelectrolyte effect and further validates the trend of increasing hydrodynamic diameters (Fig. 3a). The increase in hydrodynamic diameter was therefore not due to pCBMA chain extension. Additionally, the change in hydration layers of Lyz(5+)pCBMA matched that of free pCBMA showing that pCBMA-solvent interactions are similar in response to increased NaCl concentrations whether free in solution or attached to a protein surface (Supplementary Fig. 12). We have previously shown, through MD simulations, that poly(carboxy betaine) acrylamide chains were highly dynamic and did not interact strongly with a protein surface^3^. This is also seen qualitatively in the Lyz(5+)pCBMA conjugate snapshot (Supplementary Fig. 13) in low and high salt concentrations. Therefore, although Lyz’s surface is highly decorated with different charged amino acids, the presence of this complex interface did not affect the salt-induced changes in pCBMA conformation. The extension of polymer chains away from Lyz’s surface left much of Lyz’s surface exposed (Supplementary Fig. 13), confirming that the altered solubility of Lyz–polymer conjugates was derived from changes in Lyz’s physicochemical properties rather than polymer wrapping around the protein to mask Lyz’s surface properties.

Hydration layers were also determined for Lyz(5+)pOEGMA and free pOEGMA in increasing NaCl concentration. RDFs were consistent between free and conjugated simulations. There were two discernable hydration layers around the PEG units in pOEGMA (2.5 and 5.0 Å) and while the total number of hydration layers remained constant, the likelihood of finding a water molecule in those layers decreased with increasing NaCl concentration (Fig. 4e). Additionally, the probability of finding a Cl^−^ or Na^+^ ion near pOEGMA decreased (Fig. 4f and Supplementary Fig. 11). It is important to note that both R_g_ and level of hydration start to significantly decrease after 1.5 M salt concentration, corresponding to 36% saturated ammonium sulfate, which is the point at which almost all of Lyz(5+)pOEGMA has precipitated (Fig. 2b). Overall, the salting-out of Lyz-pCBMA conjugates was prevented because the hydration layers around the conjugate increased with increasing salt concentration. Lyz-pOEGMA conjugates collapsed and displayed a decreased hydration which could have promoted precipitation.

### Conjugate activity in 100% saturated ammonium sulfate

The newly discovered solubility of a protein in saturated salt solutions raises the interesting question of whether functional activity can be retained in this environment. A challenge in most precipitation methods is loss of protein function. We therefore determined the activities of Lyz-pCBMA conjugates containing 1, 3, and 5 polymer chains in saturated ammonium sulfate using a small molecule fluorescent substrate, 4-methylumbelliferyl β-D-N,N′,N′′-triacetylchitotrioside. There was no correlation between activity and pCBMA length in either 50 mM sodium phosphate (NaPhos) or saturated ammonium sulfate (Supplementary Table 2 and Supplementary Fig. 14). Lyz remained active after initiator attachment and pCBMA growth. Lyz(5+)pCBMA conjugates had increased activities in 100% saturated ammonium sulfate and were up to 1.6 times higher than the corresponding activities in NaPhos buffer, even at slightly lower pH (pH 6.0 versus pH 5.5). Interestingly, Lyz-initiator displayed the highest activity in ammonium sulfate (4.2 times more than in NaPhos buffer). Activities of Lyz-pCBMA conjugates with 1 and 3 initiators were also measured (Supplementary Table 2 and Supplementary Fig. 14). The conjugates remained active after polymer growth and were again, more active in 100% ammonium sulfate. Additionally, Lyz(1+) and Lyz(3+) were 1.8 and 2.4 times more active in ammonium sulfate than NaPhos, respectively. Lyz-initiator should be aggregated at 100% ammonium sulfate saturation. Aggregation typically leads to unfolding and loss of activity. The ammonium cation is highly kosmotropic, however, and stabilizes the protein structure during precipitation to keep Lyz active. The chemical structure of the initiator contains a positively charged quaternary ammonium. This could have strongly attracted sulfate anions and slightly changed the arrangement of the active site residues to strengthen the active site-substrate interaction to increase activity in ammonium sulfate^57^.

### Purification by utilizing different salting-out points

**Fig. 5:**
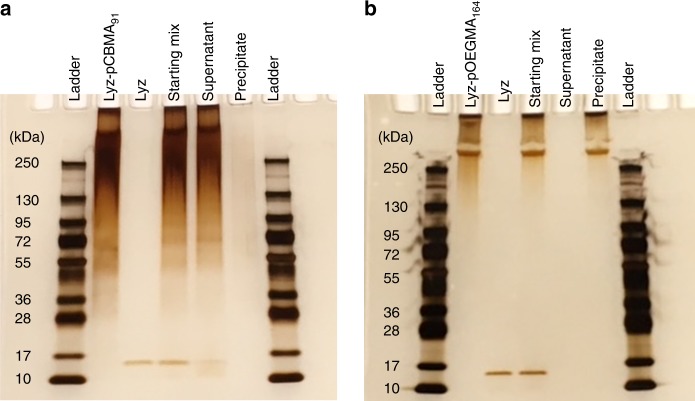
Purification using SDS-PAGE analysis. Silver stained SDS-PAGE gels to show purification of a mixture of native Lyz and **a** Lyz(5+)pCBMA DP 91 or **b** Lyz(5+)pOEGMA DP 164. Samples were mixed in a 1 to 99 volume ratio of native Lyz to conjugate (starting mix) and ammonium sulfate was added to preferentially precipitate native Lyz from Lyz-pCBMA (100% saturation) or to precipitate Lyz-pOEGMA from native Lyz (40% saturation). Supernatants and precipitates were dialyzed in deionized water to remove salt and were then concentrated back to starting concentrations using ultrafiltration prior to SDS-PAGE analysis

After verifying that ammonium sulfate did not deactivate the conjugates, the differences in solubilities were utilized to purify a mixture of conjugates and minute amounts of native Lyz (10 µg/mL). A hurdle in protein–polymer conjugate synthesis and characterization is heterogeneity. This makes characterization difficult^6^ and it also impedes accurate measurements of activity and stability since unmodified protein may remain in the sample. Various chromatography techniques are typically used to purify conjugates including size exclusion, ion exchange, or high performance reverse phase chromatography, which all require instrumentation and user-knowledge. Ammonium sulfate, however, is inexpensive and does not require high-end analytical equipment. We therefore mixed long-chained pCBMA and pOEGMA conjugates with native Lyz in a 1:99 volume ratio of native Lyz to conjugate (5 initiators), preferentially precipitated one of the species, then performed SDS-PAGE analysis of the supernatant and precipitate. Native Lyz and Lyz-pOEGMA DP 164 precipitated around 60% and 15%, respectively, while Lyz-pCBMA DP 91 did not precipitate at all. Therefore, to purify a mixture of native Lyz and Lyz-pOEGMA, ammonium sulfate was added at 40% saturation to preferentially precipitate the conjugate and to purify a mixture of native Lyz and Lyz-pCBMA, ammonium sulfate was added at 100% saturation to preferentially precipitate native Lyz. After preferential precipitation, samples were dialyzed in deionized water to remove the salt and then ultrafiltration was performed to obtain samples with the concentration of the starting mixture. Lyz(5+)pCBMA and Lyz(5+)pOEGMA gel lanes show the typical band broadening after polymer conjugation and increases in molecular mass over native Lyz (Fig. 5). Both native Lyz and Lyz(5+)pCBMA can be seen in the starting mixture, and after preferential precipitation, the band for native Lyz in the supernatant noticeably decreased (Fig. 5a). The gel was analyzed in ImageJ to compare the intensities of the native Lyz band before and after purification to estimate a final concentration of 0.003 mg/mL from a starting concentration of 0.01 mg/mL. A second round of preferential precipitation was performed on that supernatant and after another SDS-PAGE analysis, no native Lyz was detected in the supernatant (Supplementary Fig. 15).

For Lyz(5+)pOEGMA, both the conjugate and native Lyz bands can be seen in the starting mixture (Fig. 5b). After purification with 40% ammonium sulfate, the conjugate was preferentially precipitated and native Lyz was not detected in the precipitate indicating successful purification. Additionally, Image J was used to compare the band intensities of the conjugate in the starting mixture and the conjugate in the precipitate. The purification yield was estimated as 61%. Although there was a decrease in yield, the broadening of the band was decreased so that a more homogenous conjugate was purified. This method of purification based on differences in solubilities was very useful for conjugates of high modification where the salting-out point was much different than the native protein. We believe this method can be utilized for other polymer types as well.

### Effect of other charged polymers on conjugate solubility

Finally, we exemplified the range of conjugates that purification by ammonium sulfate precipitation could be useful. α-Chymotrypsin (CT)-polymer conjugates that were previously synthesized and characterized^3^ were studied. Briefly, 12 long-chained polymers of zwitterionic pCBMA (DP 112), neutral pOEGMA (DP 97), neutral/positive poly(dimethylaminoethyl methacrylate) (pDMAEMA) (DP 89), positively charged poly(quaternary ammonium methacrylate) (pQA) (DP 89), and negatively charged poly(sulfonate methacrylate) (pSMA) (DP 113) were grown from the surface of CT that had been modified with 12 neutral initiators, on average^3^. Ammonium sulfate precipitation was performed on native CT, CT-initiator, and CT-polymer conjugates (Supplementary Fig. 16). Native CT precipitated near 60% saturation and CT-initiator precipitated around 20% saturation. This is much different than the case with Lyz where native Lyz and Lyz-initiator precipitated around the same point (Supplementary Fig. 16). Lyz conjugates were synthesized with a positively charged initiator that preserved the positive charge that was previously on the amino group. CT conjugates, however, were synthesized with a neutral initiator where covalent attachment converts the positively charged amino group to a non-ionizable amide bond thereby increasing the negative to positive charge ratio on the local protein surface to increase hydrophobicity. This decrease in charge density caused the CT-initiator to precipitate at much lower ammonium sulfate concentrations. It also precipitated slowly over the range of 20–60% saturation, which again, could be potentially useful for sample fractionation to synthesize homogenous conjugates. Since the advantages of using a charged initiator were only published recently^13^, the majority of grafted-from initiators in the current literature are still uncharged and target protein amino groups^12,58,59^. Therefore, the precipitation of protein-initiator before the native protein is advantageous for separating unmodified from modified protein after the initiator reaction. As with Lyz, growth of pCBMA increased CT’s solubility up to 100% saturation while growth of pOEGMA decreased solubility to 15% saturation. Interestingly, the positively charged pQA, positively charged pDMAEMA, and negatively charged pSMA conjugates all remained soluble up to 100% saturation, as well. pDMAEMA has a pKa around 6.2 (ref. ^3^) and at pH 7, would be 15% protonated and positively charged which was enough charge to keep CT soluble. Typical polyelectrolyte polymers collapse in high salt concentrations by screening of electrostatic interactions, but remain hydrated, which would have prevented precipitation^60^. There are many other types of monomers, both commercially available and that can be synthesized in-house, that are amenable to grafting-from techniques, namely ATRP or reversible addition-fragmentation chain-transfer (RAFT) polymerization. Therefore, polymers can be specifically designed to tune the solubility of a protein–polymer conjugate to ease purification.

Overall, covalent attachment of polymers to a protein can significantly alter the protein’s solubility, which can be tuned by changing the polymer type, grafting density, and polymer length. The grafting-from approach to conjugate synthesis easily allows for each of those variables to be tuned independently. Highly charged polymers (zwitterionic, positive, and negative) increase the solubility up to 100% ammonium sulfate saturation and prevent salting-out while uncharged, amphiphilic polymers decrease solubility. Zwitterionic polymer conjugates that are soluble in 100% ammonium sulfate remain active and are stable for at least 2.5 months of storage. Experimental and simulation results showed that zwitterionic polymers, when bound to the protein surface, display the anti-polyelectrolyte effect with increasing salt concentration similar to the behavior of unbound zwitterionic polymers in salt solutions. Due to this effect, the conjugates are highly solvated at high ammonium sulfate concentrations. The differences in solubilities between conjugates and either native protein or protein-initiator can be utilized for simple purification and fractionation of heterogeneous mixtures.

The protein–polymer conjugates that were soluble in 100% ammonium sulfate (4.1 M) would fall into the category of extreme halophilic proteins that are able to survive in the harshest of salt conditions which further shows how polymer conjugation can increase the robustness of a protein to survive in non-native environments. The addition of ammonium sulfate to a protein solution can have many uses since it is kosmotropic and promotes stabilization. In general, polymer conjugation to a protein can cause structural changes and deactivation of the protein. Since ammonium sulfate has been shown to help refold misfolded/unfolded proteins, it could also be used to re-structure protein–polymer conjugates or help maintain the protein structure (prevent unfolding) throughout conjugate synthesis (initiator attachment and polymer growth). Additionally, polymer attachment has been shown to increase the thermostability of a protein. Ammonium sulfate has also been shown to increase a protein’s thermostability. Therefore, the addition of ammonium sulfate to a protein–polymer conjugate solution could potentially increase the thermostability of a protein beyond the increase provided by the polymer. Another potential application is in the purification of a target protein from cell lysate, enabled through the use of non-natural amino acids. The target protein can be genetically engineered with a specific reactive group on a non-natural amino acid. Zwitterionic polymers can then by reacted to (or grown from) the non-natural amino acid followed by ammonium sulfate precipitation. The target protein will remain soluble while all other cell lysate contaminants will precipitate. The non-natural amino acid can also be engineered to contain a reversible/cleavable group so that the zwitterionic polymer can be cleaved after purification to yield the native target protein. This approach could have significant impact in antibody purification as an alternative to Protein A chromatography. In general, we now have the ability to keep proteins soluble in high concentrations of salts by polymer conjugation which can be utilized for many new and unforeseen applications.

## Methods

### Materials

Lysozyme (Lyz) from hen egg white, α-chymotrypsin (CT) from bovine pancreas, glycine, copper(II) chloride (Cu(II)Cl), sodium ascorbate (NaAsc), 1,1,4,7,10,10-hexamethyltriethylenetetramine (HMTETA), and poly(ethylene glycol) methyl ether methacrylate (OEGMA_500_) were purchased from Sigma-Aldrich (St. Louis, MO). 3-[[2-(Methacryloyloxy)ethyl]dimethylammonio] propionate (CBMA) was purchased from TCI America. HMTETA was purified using a basic alumina column. Pierce silver stain kit was purchased from ThermoFisher. SDS-PAGE gels (4–15% Mini-PROTEAN TGX precast gels) were purchased from Bio-Rad. All other chemicals were used without further purification and were purchased from Sigma Aldrich unless otherwise stated. The positively charged ATRP initiator was prepared as previously described^13^. Dialysis tubing for purification was purchased from Spectra/Por, Spectrum Laboratories Inc., CA.

### Instrumentation

Ultraviolet-visible (UV-VIS) spectrophotometry (Lambda 45, PerkinElmer) was used to determine protein concentrations from bicinchoninic acid (BCA) assays. Number average (*M*_n_), weight average (*M*_w_), and dispersity (*Đ*) of polymers (cleaved and free) were determined by gel permeation chromatography (GPC) (Waters 2695 Series) with a data processor, three columns (Waters Ultrahydrogel Linier, 250 and 500), and a refractive index detector using a running buffer of Dulbecco’s Phosphate Buffered Saline with 0.02 wt% sodium azide at a flowrate of 1.0 mL/min. Calibration was performed using Pullulan standards (Polymer Standards Service, Amherst, MA). Matrix-assisted laser desorption/ionization time-of-flight mass spectrometry (MALDI-ToF MS) data was acquired on a Perseptive Biosystems Voyager, Elite MALDI-ToF spectrometer located in the Center for Molecular Analysis at Carnegie Mellon University. Dynamic light scattering (DLS) hydrodynamic diameters were measured on a Malvern Zetasizer nano-ZS located in the Department of Chemistry at Carnegie Mellon University. Ammonium sulfate precipitation analysis and enzymatic activities were measured on a Synergy H1 Multi-Mode Plate Reader (BioTek Instruments, Winooski, VT).

### ATRP initiator modifications (1, 3, 5) on Lyz

To synthesize Lyz with an average of 1 initiator modification (Lyz(1+)), 100 mg (0.007 mmol Lyz, 0.049 mmol NH_2_) of native Lyz was dissolved in 20 mL of 0.1 M sodium phosphate buffer, pH 8. 25 mg of positively charged ATRP initiator (0.049 mmol, 1 equivalent against the number of NH_2_ groups) was dissolved in 100 μL of DMSO. The dissolved initiator was added to the Lyz solution and stirred at 4 °C for 2 h. Lyz-initiator was then purified by dialysis (8 kDa MWCO) against deionized water at 4 °C and was subsequently lyophilized.

To synthesize Lyz with an average of 3 initiator modifications (Lyz(3+)), 150 mg (0.01 mmol Lyz, 0.073 mmol NH_2_) of native Lyz was dissolved in 29 mL of 0.1 M sodium phosphate buffer, pH 8. 114 mg (0.221 mmol, 3 equivalents against the number of NH_2_ groups) of positively charged initiator, dissolved in 1 mL DMSO, was added to the Lyz solution and stirred for 2 h at 4 °C. Initiator modified Lyz was purified by dialysis as described above and was subsequently lyophilized.

To synthesize Lyz with an average of 5 initiator modifications (Lyz(5+)), 500 mg (0.035 mmol Lyz, 0.245 mmol NH_2_) of Lyz was dissolved in 100 mL of 0.1 M sodium phosphate buffer, pH 8. 631 mg of positively charged initiator (1.22 mmol, 5 equivalents against the number of NH_2_ groups) was dissolved in 1 mL DMSO and was then added to the Lyz solution. The reaction solution was stirred at 4 °C for 2 h. Initiator modified Lyz was purified by dialysis as described above and was subsequently lyophilized.

### MALDI-ToF

Initiator modified Lyz (1 mg/mL) or native Lyz (1 mg/mL) was mixed with MALDI matrix (10 mg sinapinic acid, 250 μL of 0.1% trifluoroacetic acid and 250 μL of 50% acetonitrile) in 1:1 ratio. Two microliters of mixed sample was loaded onto a sterling silver MALDI target plate. MALDI-TOF MS measurements were recorded using a Perseptive Voyager STR MS with a nitrogen laser (337 nm) and 20 kV accelerating voltage with a grid voltage of 90%. A total of 500 laser shots covering the complete spot were accumulated for each spectrum. Cytochrome C, apomyoglobin, and aldolase were used as calibration samples. The average number of initiator attached to Lyz was determined by taking the difference in peak m/z vales between native Lyz and Lyz-initiators and dividing by the mass of the reacted initiator (without NHS group) (321 Da).

### ATRP from Lyz-initiator

20 mg of Lyz(1+) (1.4 μmol ATRP initiator groups) and 7.8 mg CBMA for target DP of 25, 62 mg CBMA for target DP of 200, 17 mg OEGMA for target DP of 25, and 136 mg OEGMA for target DP of 200 were dissolved in 1120 µL of 0.1 M sodium phosphate, pH 8. Lyz(1+) and monomer solutions were bubbled under argon for ~7 min. Concurrently, 336 μL of 50 mM Cu(II)Cl in deionized water was bubbled under argon in a separate flask for 2 min. Next, 16.8 μL of 100 mM sodium ascorbate was added to the Cu(II)Cl solution. After that, 5.3 μL of HMTETA was added to reduced Cu(II) to Cu(I) and the solution was bubbled for an additional minute. Next, 280 μL of the Cu/ligand solution was added to the Lyz-initiator/monomer solution using a syringe and the sealed solution was stirred for 1 h. The reaction was stopped by exposure to air and then the conjugates were purified using dialysis (8 kDa MWCO) against deionized water for 24 h and were subsequently lyophilized.

30 mg of Lyz(3+) (6.4 μmol initiator groups) was dissolved in 5760 μL of 0.1 M sodium phosphate, pH 8. 37 mg CBMA or 81 mg OEGMA (target DP 25) and 295 mg CBMA or 644 mg OEGMA (target DP 200) were added to Lyz(3+) and bubbled for 15 min under argon. In a separate flask, 768 μL of 100 mM Cu(II)Cl solution was bubbled under argon for 2 min. Next, 77 μL of 100 mM sodium ascorbate solution was added to the bubbling Cu(II)Cl solution. Then, 25 μL HMTETA ligand was added to reduced Cu(II) to Cu(I) and the solution was bubbled for an additional minute. Next, 640 μL of the Cu/ligand solution was transferred via syringe to the Lyz-initiator/monomer solution. The polymerization was stopped by exposure to air after 1 h of stirring. The conjugates were purified using dialysis (8 kDa MWCO) against deionized water for 24 h and were subsequently lyophilized.

In all, 30 mg (9.5 μmol initiator groups) of Lyz(5+) was dissolved in 18 mL of 0.1 M sodium phosphate, pH 8. 54 mg, 109 mg, 217 mg, 326 mg, and 434 mg CBMA (for the target DP of 25, 50, 100, 150 and 200, respectively) were added to Lyz(5+) and were bubbled under argon for 45 min. In a separate flask, 2.4 mL of 50 mM Cu(II)Cl solution was bubbled for 10 min. Next, 114 μL of 100 mM sodium ascorbate was added to reduce Cu(II) to Cu(I) and then 37 μL of HMTETA ligand was added. After that, 2 mL of the Cu/ligand solution was added to Lyz-initiator/CBMA solution. The reaction was stopped upon exposure to air after 1 h for stirring and the Lyz(5+)pCBMA conjugates were purified using dialysis (8 kDa MWCO) against deionized water for 24 h and were subsequently lyophilized.

32 mg (10 μmol initiator groups) of Lyz(5+) and 126 mg, 252 mg, 505 mg, 758 mg and 1010 mg OEGMA (for the target DP of 25, 50, 100, 150 and 200, respectively) were dissolved in 9 mL of 0.1 M sodium phosphate, pH 8. The Lyz(5+)/monomer solution was bubbled for 30 min under argon. In a separate flask, 1.2 mL of 100 mM Cu(II)Cl solution was bubbled for 10 min. Next, 120 μL of 100 mM sodium ascorbate was added to reduce Cu(II) to Cu(I) and then 39 μL of HMTETA ligand was added. Then, 1 mL of the Cu/ligand solution was added to the Lyz(5+)/OEGMA solution. The reaction was stopped upon exposure to air after 1 h or stirring and the Lyz(5+)pOEGMA conjugates were purified using dialysis (8 kDa MWCO) against deionized water for 24 h and were subsequently lyophilized.

### Free polymer synthesis

4.7 mg (0.92 mM final concentration) of neutral initiator (synthesized as previously described^41^) and 442 mg CBMA (target DP 100) or 894 µL OEGMA (target DP 100) were dissolved in 20 mL of deionized water and were bubbled under argon for 30 min. In a separate flask, 78 mg of Cu(II)Cl in 3 mL of deionized water was bubbled under argon. Next, 573 µL of a 20 mg/mL sodium ascorbate solution was added to reduce Cu(II) to Cu(I) and the solution was bubbled for 5 min before adding 186 µL of HMTETA, followed by additional bubbling for 1 minute. Next, 1 mL of the Cu/ligand solution was transferred to the initiator/monomer solution via syringe and the sealed flask was stirred for 1 h at 25 °C. The final concentrations in the ATRP reaction were 92 mM monomer, 0.92 mM initiator, 9.2 mM Cu(II) (reduced), 11 mM HMTETA, and 0.92 mM NaAsc. The polymerization was stopped by exposure to air and the polymers were purified by dialysis (1 kDa MWCO) against deionized water for 24 h at 25 °C. Purified polymers were then lyophilized and analyzed by GPC for molecular masses and dispersities.

### BCA assay to determine protein concentration

To determine the protein content in the conjugates, 1–3 mg/mL of Lyz–polymer samples were prepared in deionized water. 25 μL of the sample was then mixed with 1 mL of BCA solution (50:1 vol:vol of BCA and Cu(II)SO_4_) and incubated at 60 °C for 15 min. The absorbance was recorded at 562 nm. Protein concentration was determined against a standard curve of native Lyz (0.8-0.012 mg/mL) in deionized water. Lyz–polymer conjugates molecular masses and degree of polymerizations were estimated as previously described^41^.

### Dynamic light scattering to determine conjugate size in PBS

Hydrodynamic diameters of Lyz samples were determined on a Malvern Zetasizer nano-ZS. Lyz samples (native, initiator modified, and polymer modified) were dissolved at 1 mg/mL in 0.1 M sodium phosphate buffer, pH 8. Samples were filtered using a 0.45 µM cellulose acetate syringe filter and measured three times (15 runs per measurement). Reported values are number distribution hydrodynamic diameters.

### Acid hydrolysis and GPC

In all, 10–15 mg of Lyz–polymer conjugates were dissolved in hydrolysis tubes using 6 N HCl (5 mL). After three repetitions of freeze-pump-thaw cycles, the samples were place in an oil bath at 110 °C under vacuum for 20 h. Cleaved polymers were purified by dialysis (1 kDa MWCO) against deionized water and were then lyophilized. Cleaved polymers were analyzed by GPC for molecular masses and dispersities using pullulan standards as previously described.

### Ammonium sulfate precipitation

Native protein, protein-initiators, and protein-polymers were dissolved at 2 mg/mL protein concentration (starting volume was 1 mL) in 50 mM NaPhos buffer, pH 7. The initial concentrations of protein in the samples were measured by the absorbance 280 nm using a Synergy H1 plate reader. Absorbance values were converted to concentrations based on a standard curve of native protein (0 to 2 mg/mL). Solid amounts of ammonium sulfate were added to the solutions to reach the desired percent saturation as calculated from EnCor Biotechnology’s online calculator at 25 °C (http://www.encorbio.com/protocols/AM-SO4.htm). After each ammonium sulfate addition, samples were vortexed to ensure full dissolution of the ammonium sulfate. The samples were then allowed to sit on the benchtop for 15 min followed by centrifugation at 16,800*×g* for 20 min to pellet any precipitated protein. The protein concentration in the supernatant was measured in triplicate by the absorbance at 280 nm. The supernatant used to determine protein concentration was place back into the sample and the next solid mass of ammonium sulfate was added. The process of mixing, sitting, centrifuging, and measuring protein concentration was repeated after each ammonium sulfate addition until 100% saturation (4.1 M) was reached. The addition of ammonium sulfate increased the solution volume to 1.42 mL at 100% saturation.

Ammonium sulfate was also performed for native protein in the presence of free pCBMA or pOEGMA. In this case, native Lyz was dissolved at 2 mg/mL (1 mL starting volume) in 50 mM NaPhos buffer, pH 7. Lyophilized pCBMA or pOEGMA was added to match the amount (by mass), as estimated from the BCA results, of polymer present during the precipitation experiment of Lyz(5+)pCBMA DP 91 and Lyz(5+)pOEGMA DP 164. The process of ammonium sulfate precipitation was then carried out as previously described.

### Dynamic light scattering for size in ammonium sulfate

Native protein, protein-initiators, and protein-polymers were dissolved at 1 mg/mL protein concentration (starting volume was 1 mL) in 50 mM NaPhos buffer, pH 7. Solutions were filtered using a 0.45 µM cellulose acetate syringe filter. The process used for ammonium sulfate precipitation, as described above, was repeated, but instead of measuring protein concentration in the supernatant, the hydrodynamic diameters were measured in triplicate (15 runs per measurement). Hydrodynamic diameters were measured at increasing ammonium sulfate concentrations until 100% saturation was reached. The changes in solution refractive index^61^, dielectric constant^62^, and viscosity^63^ with increasing salt did not affect the hydrodynamic diameter output.

### Dynamic light scattering to measure size stability

Lyz(5+)pCBMA DP 14 and DP 91 were dissolved at 1 mg/mL in 50 mM NaPhos buffer, pH 7. 0.77 mg of solid ammonium sulfate was added and dissolved to reach 100% saturation. Samples were filtered using a 0.45 µM cellulose acetate syringe filter. Immediately after filtering, hydrodynamic diameters were measured over 6 h, then again after 1 week, 2 weeks, and 2.5 months. Number and volume distributions were recorded from 15 scans per measurement.

### Dynamic light scattering to measure size reversibility

Lyz(5+)pCBMA DP 91 was dissolved in 100% saturated ammonium sulfate at 1 mg/mL and the hydrodynamic diameter was measured. The sample was then diluted to 50% saturation (0.5 mg/mL) and 25% saturation (0.25 mg/mL) and hydrodynamic diameters were measured after each dilution as previously described. Size reversibility was also tested by cycling between 50% and 100% saturation. Lyz(5+)pCBMA DP 91 was dissolved in 50% saturated ammonium sulfate at 1 mg/mL. The hydrodynamic diameter was measured and then solid ammonium sulfate was added to reach 100% saturation, followed by another hydrodynamic diameter measurement. The solution was then diluted to 50% saturation again (0.5 mg/mL), measured by DLS, then ammonium sulfate was added to reach 100% saturation again. This process was repeated one more time for a total of 3 complete cycles. Hydrodynamic diameters were measured as described previously.

### Atomistic MD simulations

Lyz’s crystallographic structure (PDB ID: 1AKI) was used to build the native protein structure. The reduce code in Ambertools^64^ was used to determine amino acid protonation states. The structures of free pCBMA (DP 18) and free pOEGMA (DP 25) were generated using the PySimm^65^ software package’s forcefield assisted linear self- avoiding random walk. The initiator structure was the positively charged ATRP initiator used experimentally in this study. Initiator-polymer structures were attached to the NZ atoms of Lyz at five sites: K1, K13, K33, K97, K116, which were determined from a rules-based prediction^56^. Lyz was modeled using the CHARMM C36m force field and initiators/polymers were modeled using analog parameters from CGenFF^66–68^. Topology files were generated using the psfgen tool within VMD software^69^. TIP3P water model, as implemented in NAMD, was used for solvation with a buffering distance of 14 Å. The system was neutralized by the addition of Na^+^ and Cl^−^ counterions. NaCl was additionally added at increasing molar concentrations (0.0, 0.15, 0.3, 1.0, 1.5, 2.0, 2.5, 3.0, and 5.0). The CUDA accelerated namd 2.13 (ref. ^70^) was used to perform the atomistic MD simulations which was available in the high performance cluster, hipergator2, at the University of Florida.

The conjugates were subjected to gradient energy minimizations over 10,000 steps while restraining the protein. The systems were further minimized for an additional 10,000 steps without restraint. Next, the conjugates were heated to 310.15 K in 20 ps intervals and 50 K increments using a NVT ensemble followed by 500 ps of additional simulation. After that, the NPT ensemble was applied to each system at 1 bar with a nonbonded cut-off of 12 Å and a force switching at 10 Å. Particle Mesh Ewald summation was used for long-range electrostatic interactions. Bond lengths involving hydrogen atoms were constrained using a 2 fs time step with the SETTLE algorithm. Simulations were performed for 500 ns for pCBMA and 200 ns for pOEGMA and analyses were performed using in-house VMD tcl scripts. In all, 200 ns was enough simulation time to see the collapse of pOEGMA for a relatively long time.

### Enzymatic activity assay

Enzymatic activities of native Lyz, Lyz-initiators, and Lyz-pCBMA conjugates were measured using 4-Methylumbelliferyl β-D-N,N′,N′′-triacetylchitotrioside, a small molecule fluorescent substrate (*λ*_ex_ = 360 nm, *λ*_em_ = 455 nm). Lyz solutions were prepared at 1 mg/mL (Lyz concentration) in 50 mM NaPhos, pH 6.0. The substrate was dissolved in DMSO at 5 mg/mL (6.4 mM). To start the reaction, 29 µL of the 1 mg/mL Lyz solutions (2 µM final concentration) was added with 8 µL of substrate solution (50 µM final concentration) and 963 mL of either 50 mM NaPhos (pH 6.0) or 100% saturated ammonium sulfate. Reactions were incubated at 37 °C in a water bath. At increasing time points over 4 h, 50 µL of the reaction mixture was mixed with 150 µL of stop buffer (100 mM glycine-NaOH, pH 11) in a 96-well plate. The fluorescence intensities (relative fluorescence units: RFU) were then measured in triplicate. Reaction rates were corrected by blanks of the substrate (8 µL) in either NaPhos, pH 6.0 and 100% saturated ammonium sulfate (992 µL). RFU versus reaction time plots were fit to linear regressions in GraphPad.

### Purification and SDS-PAGE gel analysis (pCBMA and pOEGMA)

Native Lyz and Lyz(5+)pOEGMA, and Lyz(5+)pCBMA DP 91 were prepared at 1 mg/mL in deionized water. Native Lyz and conjugates were mixed at a 1:99 volume ratio (10 µL native Lyz and 990 µL conjugate). Solid ammonium sulfate was added to reach 100% saturation for pCBMA (0.77 g) or 40% saturation for pOEGMA (0.25 g). The mixtures were allowed to sit for 1 h on the benchtop, followed by centrifugation at 16,800*×g* for 1 h. The supernatants were aspirated and the precipitates were re-dissolved in 1 mL of deionized water. Supernatants and precipitates were dialyzed in deionized water to remove ammonium sulfate for 24 h at 4 °C. Ultrafiltration (3 kDa MWCO) was performed on dialyzed samples to concentrate them back to starting concentrations. SDS-PAGE analysis was performed on native Lyz, Lyz(5+)pCBMA DP 91, Lyz(5+)pOEGMA DP 164, supernatants, precipitates, the starting mixture (prior to salt addition), and standards. Twenty-five microliter of samples were mixed with 25 µL of sample buffer (190 µL of 2X Lamaelli sample buffer with 10 µL of 2-mercaptoethanol). Samples were heated at 95 °C for 10 min in an oil bath. Running buffer was composed of 1X Tris/Glycine/SDS buffer. Samples (20 µL or 10 µL of ladder) were loaded into the wells of a 4–15% precast gel and electrophoresis was run at 100 V, 4 W, 40 mA for 40 min. Gels were then silver stained following the protocol provided by the Pierce Silver Stain kit. Uncropped and unprocessed gels are provided in Supplementary Fig. 17.

### Chymotrypsin–polymer synthesis and characterization

Chymotrypsin (CT)-polymers that were previously synthesized and characterized^3^ were used in the current study for ammonium sulfate precipitation analysis. Briefly, CT was modified with 12 neutral initiators and long-chained polymers of zwitterionic poly(carboxybetaine methacrylate) (pCBMA), neutral poly(oligoethylene glycol methacrylate) (pOEGMA), neutral to positive poly(dimethylaminoethyl methacrylate) (pDMAEMA), positive poly(quarternary ammonium meth- acrylate) (pQA), or negative poly(sulfonate methacrylate) (pSMA) were grown from the surface of CT-neutral initiator using ATRP. Conjugates were characterized with a BCA assay and dynamic light scattering. Additionally, acid hydrolysis was performed to cleave polymers followed by GPC analysis as described above.

### Reporting summary

Further information on research design is available in the Nature Research Reporting Summary linked to this article.

## Supplementary information

Supplementary Information

Description of Additional Supplementary Files

Supplementary Movie 1

Reporting Summary

## Data Availability

All relevant data is included in the paper, Supplementary Information, or may be available from the author upon reasonable request.
