# Supplementary material for: Transforming protein-polymer conjugate purification by tuning protein solubility
Source: Nat Commun. 2019 Oct 17;10:4718. doi: 10.1038/s41467-019-12612-9 (PMC6797786; doi:10.1038/s41467-019-12612-9)
Supplement: Supplementary file 1 — Supplementary Information [file 41467_2019_12612_MOESM1_ESM.pdf]

Supplementary Information

Transforming protein-polymer conjugate purification by tuning protein solubility

Stefanie L. Baker,[1,2] Aravinda Munasinghe,[3,4,5] Bibifatima Kaupbayeva,[2,6] Nin Rebecca Kang,[1,7] Marie Certiat,[3,8] Hironobu Murata,[2] Krzysztof Matyjaszewski,[2,9] Ping Lin,[3] Coray M. Colina,[3,4,5,10] Alan J. Russell[*1,2,7]

[1]Department of Biomedical Engineering, Scott Hall 4N201, Carnegie Mellon University, 5000 Forbes Avenue, Pittsburgh, PA 15213, United States

[2]Center for Polymer-Based Protein Engineering, Carnegie Mellon University, 5000 Forbes Avenue, Pittsburgh, PA 15213, United States

[3]Department of Chemistry, 354 Leigh Hall, University of Florida, Gainesville, FL 32611, United States

[4]George and Josephine Butler Polymer Research Laboratory, University of Florida, Gainesville, Florida 32611, United States

[5]Center for Macromolecular Science and Engineering, University of Florida, Gainesville, Florida 32611, United States

[6]Department of Biological Sciences, Carnegie Mellon University, 4400 Fifth Avenue, Pittsburgh, PA 15213, United States

[7]Department of Chemical Engineering, Carnegie Mellon University, 5000 Forbes Avenue, Pittsburgh, PA 15213, United States

[8]Université Paul Sabatier, 118 Route de Narbonne, F-31062 Toulouse, Toulouse, France

[9]Department of Chemistry, Carnegie Mellon University, 4400 Fifth Avenue, Pittsburgh, PA 15213, United States

[10]Department of Materials Science and Engineering, University of Florida, Gainesville, FL 32611, United States

Supplementary Figures

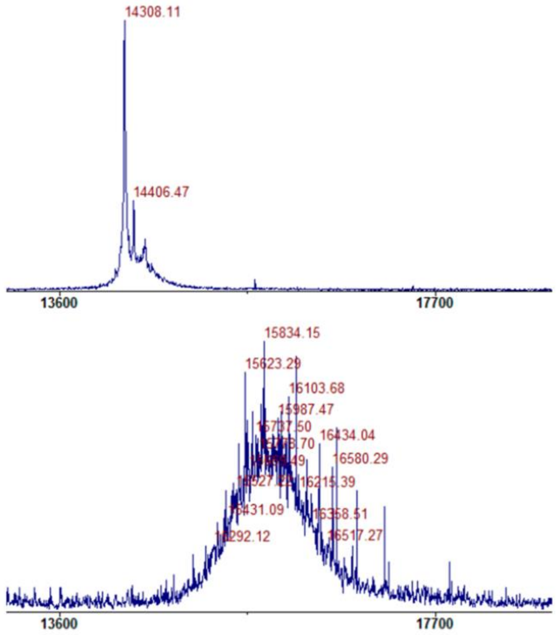

Supplementary Figure 1. MALDI-ToF spectra of native Lyz (top) and Lyz-initiator (bottom). The number of attached initiators was calculated by the difference in m/z between Lyz-initiator and native Lyz divided by the mass of the initiator (321 Da). The average number of attached initiators was 4.8.

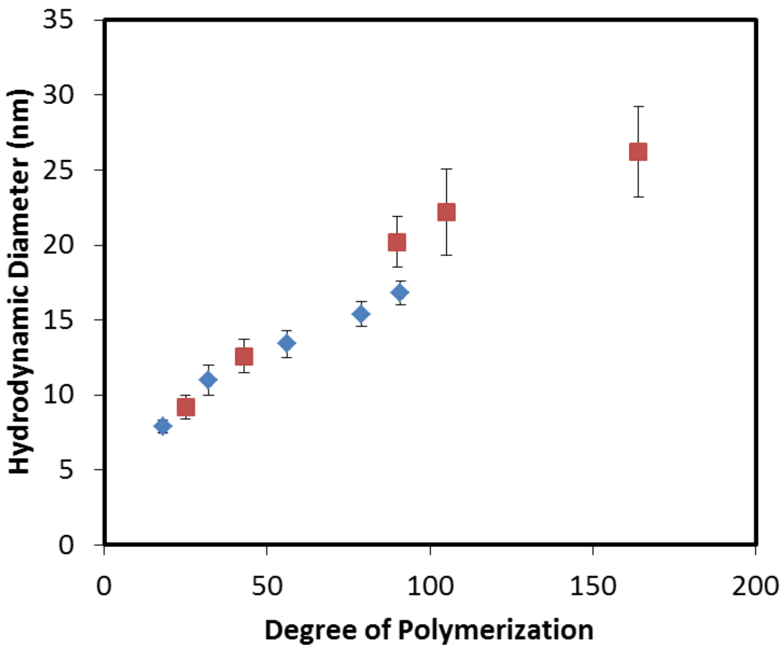

Supplementary Figure 2. Dynamic light scattering hydrodynamic diameters, by number distribution, for Lyz(5+)pCBMA (blue diamonds) and Lyz(5+)pOEGMA (red squares) conjugates of increasing polymer length (DP).

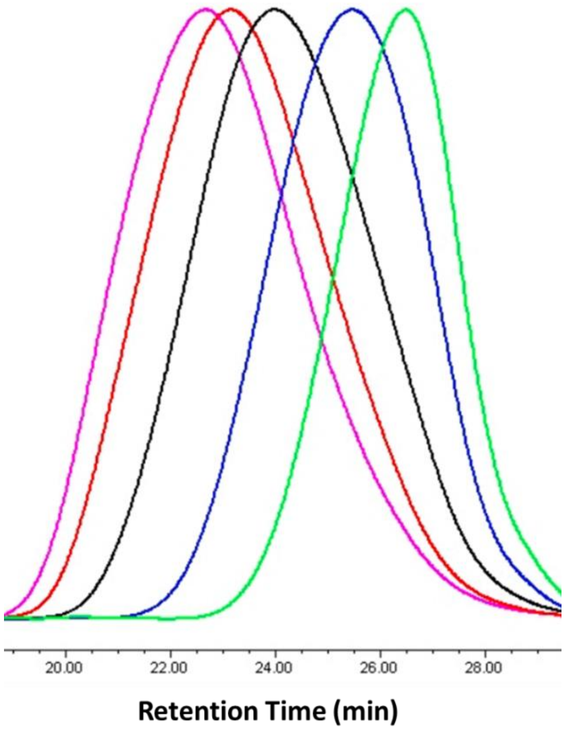

Supplementary Figure 3. Gel permeation chromatography spectra of cleaved pCBMA from conjugates. Polymers were cleaved by acid hydrolysis (6N HCl) at 110 °C under vacuum overnight and then dialyzed in deionized water. Polymers increased in molecular mass as DP increased. DP 18 (green), DP 32 (blue), DP 56 (black), DP 79 (red), DP 91 (pink).

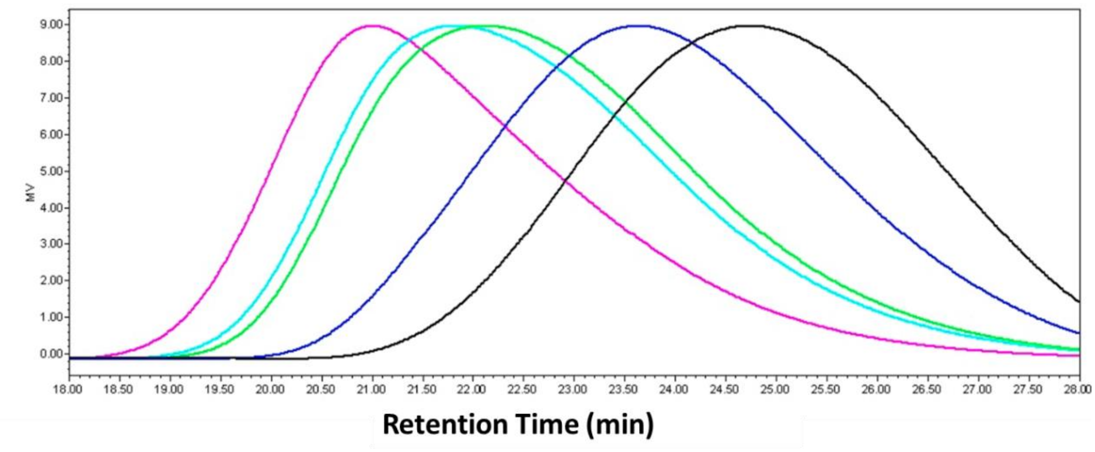

Supplementary Figure 4. Gel permeation chromatography spectra of cleaved pOEGMA from conjugates. Polymers were cleaved by acid hydrolysis (6N HCl) at 110 °C under vacuum overnight and then dialyzed in deionized water. Polymers increased in molecular mass as DP increased. DP 25 (black), DP 43 (blue), DP 90 (green), DP 105 (cyan), DP 164 (pink).

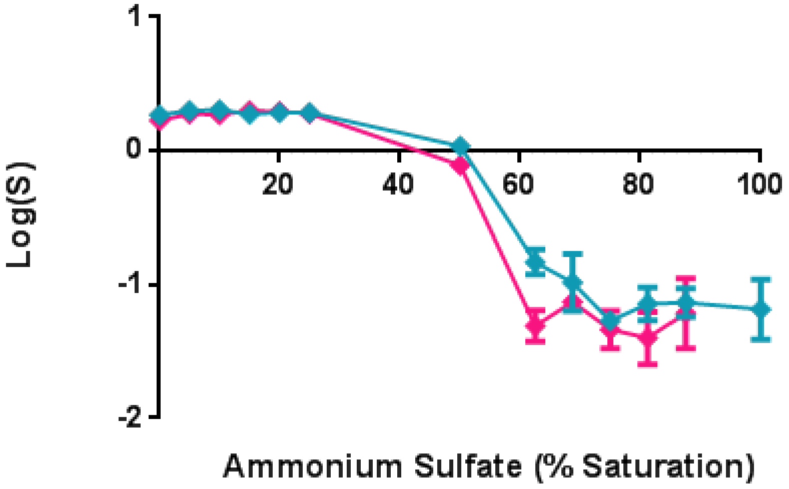

Supplementary Figure 5. Ammonium sulfate precipitation of free native Lyz in solution with free pCBMA (cyan) or pOEGMA (pink). The amount of free polymer added was the same amount of polymer that was present in the Lyz-pCBMA DP 91 or Lyz-pOEGMA DP 164 samples during the conjugate ammonium sulfate precipitation experiment.

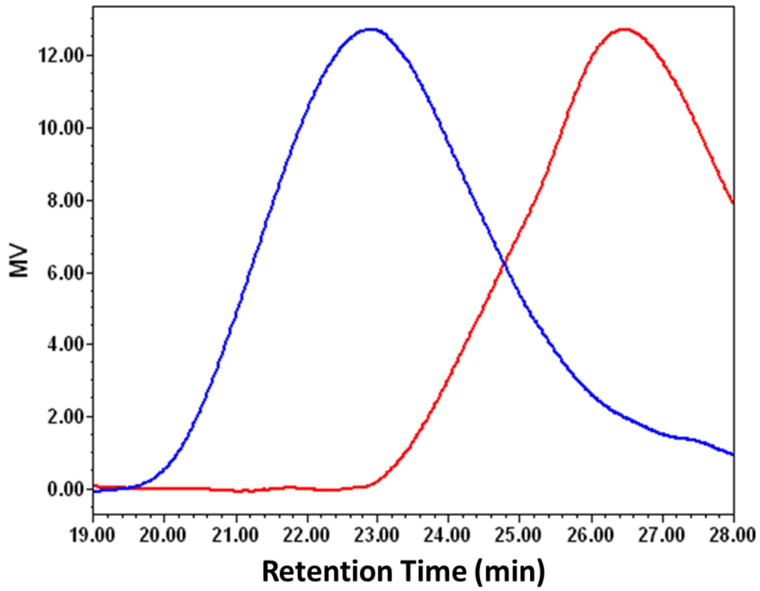

Supplementary Figure 6. Gel permeation chromatography spectra of free pCBMA (red) and pOEGMA (blue).

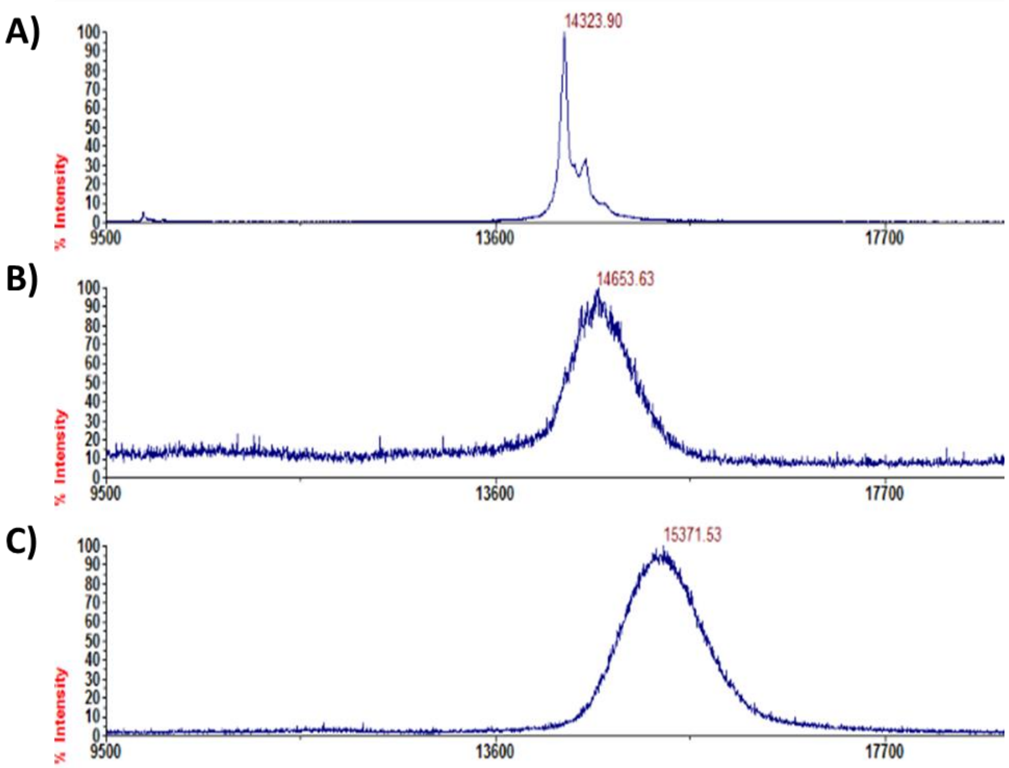

Supplementary Figure 7. MALDI-ToF spectra of **A)** native Lyz, **B)** Lyz(1+), and **C)** Lyz(3+).

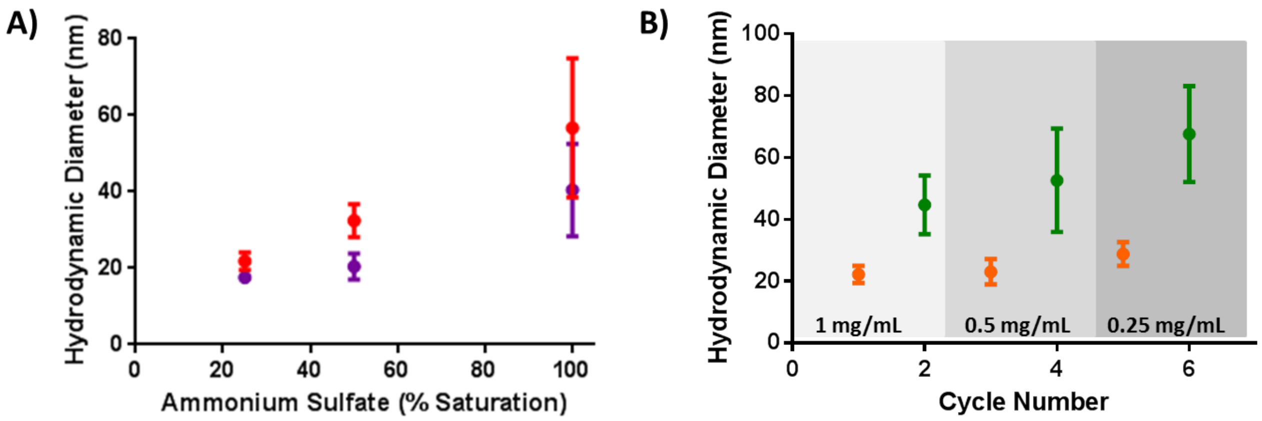

Supplementary Figure 8. Hydrodynamic diameters (number distribution averages and errors) of Lyz(5+)pCBMA DP 91 in **A)** increasing (red circle) or decreasing (purple circle) ammonium sulfate concentrations and **B)** cycling between 50% (orange circle) and 100% saturation (green circle) over 3 complete cycles. This data shows that the change in hydrodynamic diameter with ammonium sulfate concentration is reversible.

A)

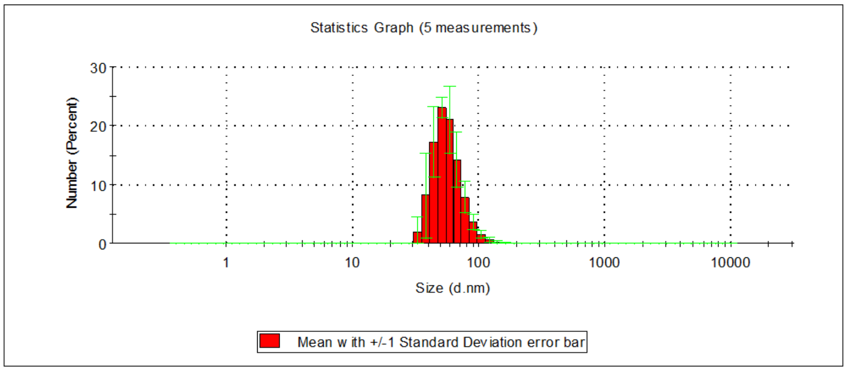

B)

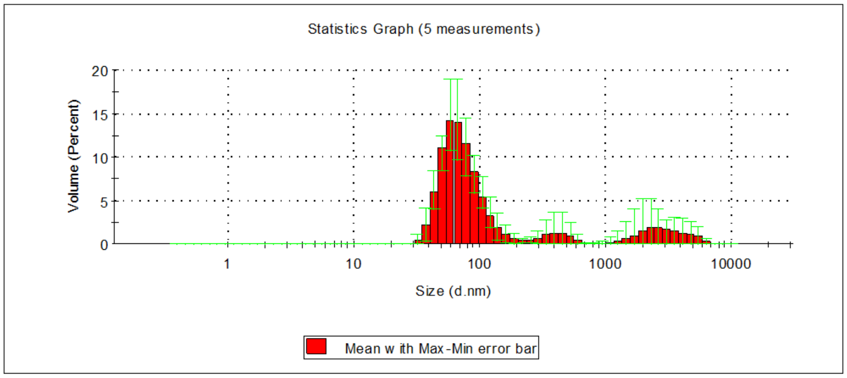

C)

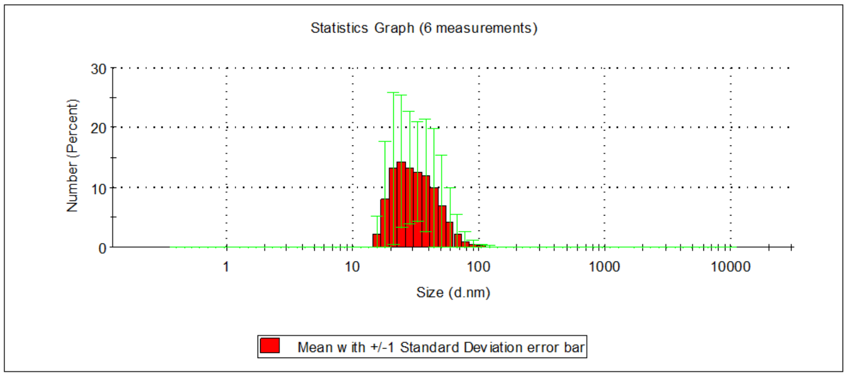

D)

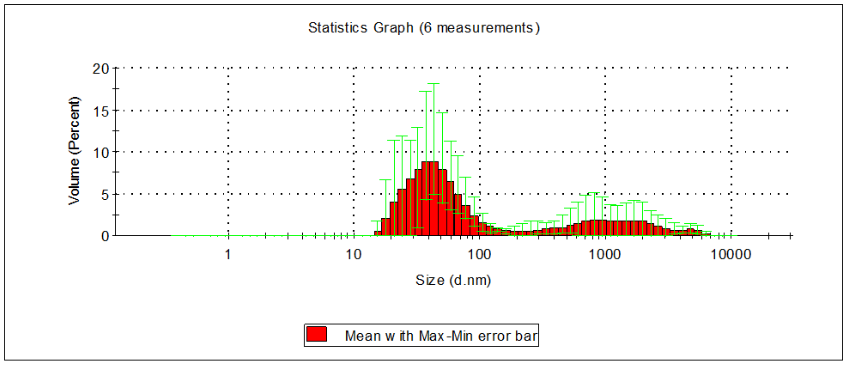

Supplementary Figure 9. Hydrodynamic diameters in 100% ammonium sulfate saturation of Lyz(5+)pCBMA DP 18 by **A)** number distribution and **B)** volume distribution and DP 91 by **C)** number distribution and **D)** volume distribution after storage for 2.5 months. Multimodal peaks are present in volume distributions indicating micro-aggregation.

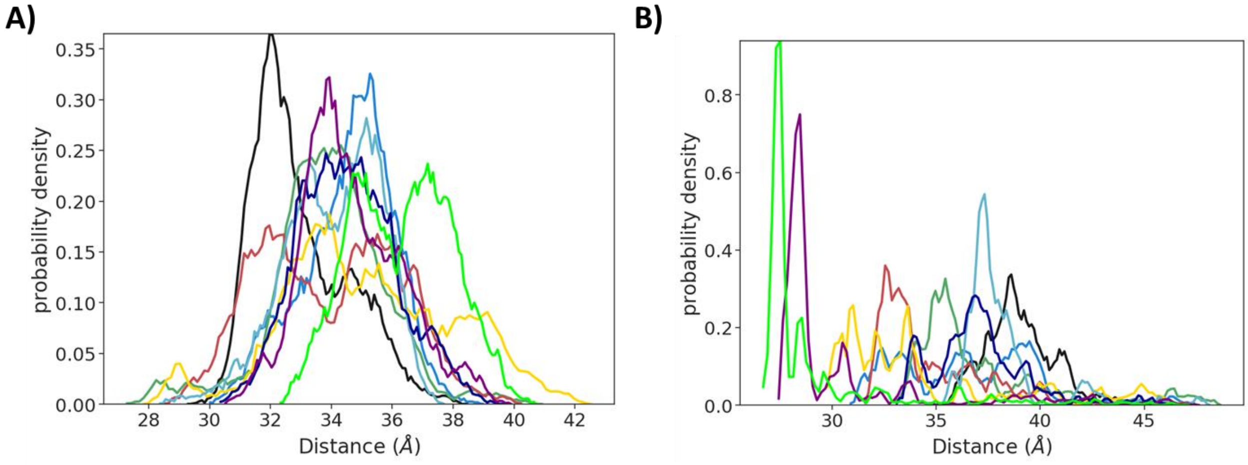

Supplementary Figure 10. R_g of **A)** Lyz(5+)pCBMA and **B)** Lyz(5+)pOEGMA in increasing NaCl: 0.0 M (black), 0.15 M (blue), 0.3 M (green), 1.0 M (red), 1.5 M (yellow), 2.0 M (light blue), 2.5 M (dark blue), 3.0 M (purple), and 5.0 M (lime green).

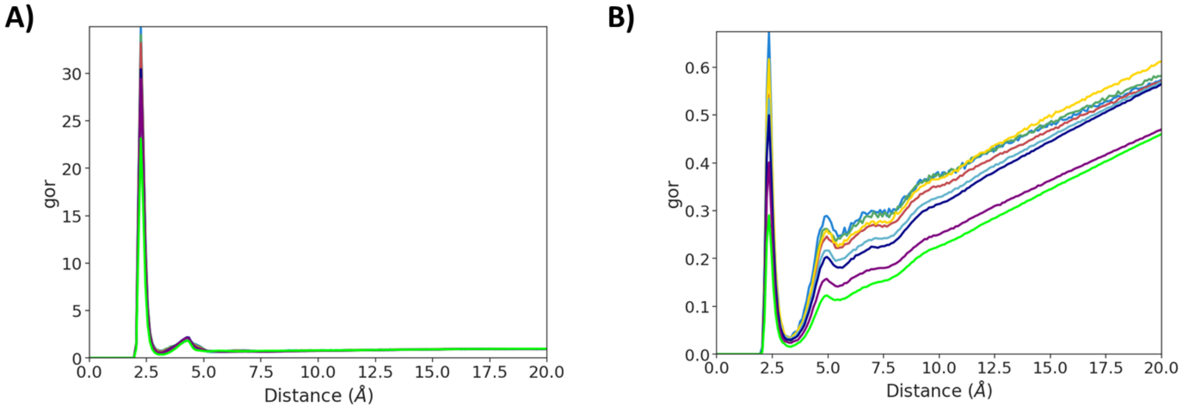

Supplementary Figure 11. Radial distribution function analyses between Na^{+} ions and O^{-} atoms of **A)** Lyz(5+)pCBMA and PEG's of **B)** Lyz(5+)pOEGMA. NaCl: 0.15 M (blue), 0.3 M (green), 1.0 M (red), 1.5 M (yellow), 2.0 M (light blue), 2.5 M (dark blue), 3.0 M (purple), and 5.0 M (lime green).

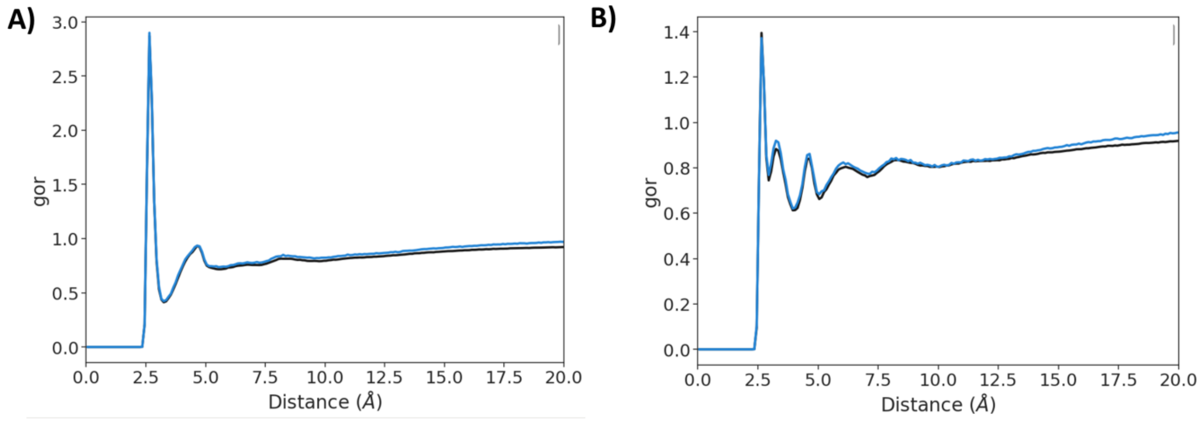

Supplementary Figure 12. Radial distribution function analyses between water molecules and O^- atoms of pCBMA at **A)** 0.15 M NaCl and **B)** 5.0 M NaCl: Lyz(5+)pCBMA (black) and free pCBMA (blue). RDF analyses are similar whether pCBMA is free in solution or bound to a protein surface.

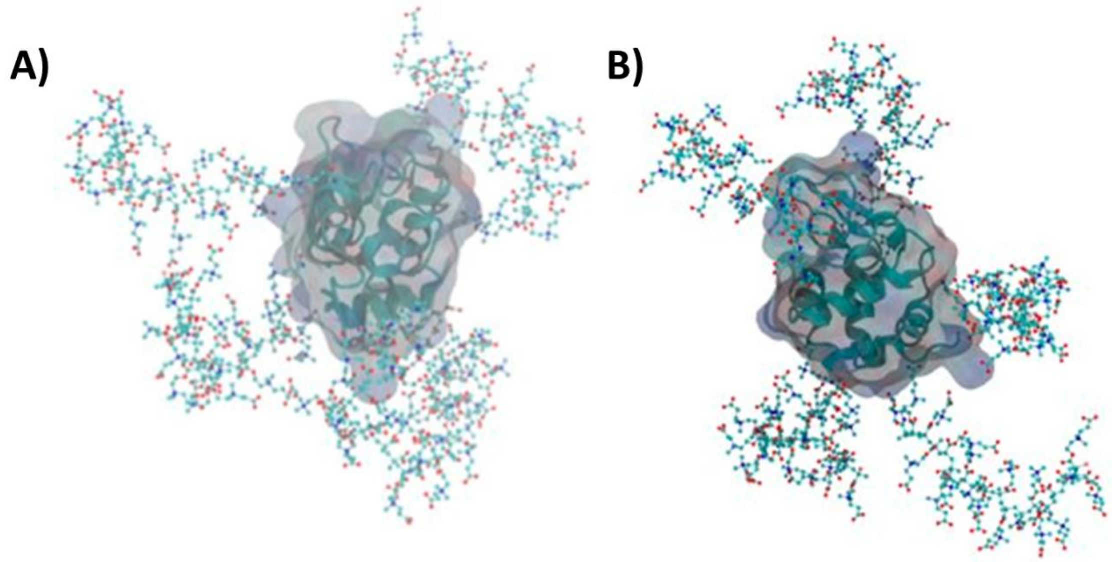

Supplementary Figure 13. Snapshot of Lyz(5+)pCBMA DP 18 during the MD simulation showing extension of polymer chains away from the protein surface and high degrees of protein surface exposure in **A)** 0.0 M and **B)** 5.0 M NaCl.

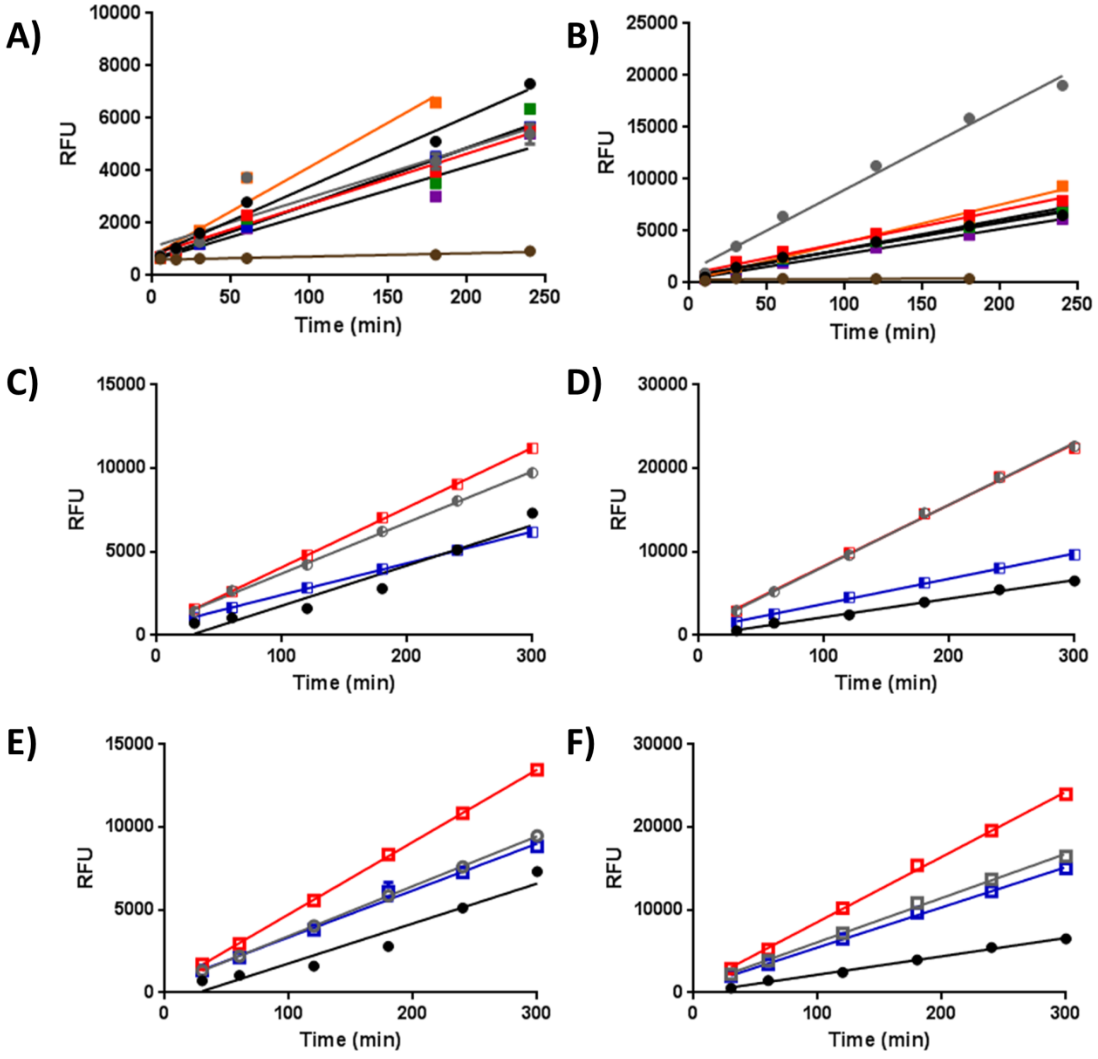

Supplementary Figure 14. Enzymatic reaction rates of Lyz-pCBMA conjugates in 50 mM NaPhos buffer (pH 6.0) (1st column) and 100% saturated ammonium sulfate (pH 5.5) (2nd column). Conjugates with 5 initiators in **A)** NaPhos and **B)** 100% ammonium sulfate for native Lyz (black circle), Lyz(5+) (gray circle), DP 18 (red square), DP 32 (orange square), DP 56 (green square), DP 79 (blue square), and DP 91 (purple square). Conjugates with 3 initiators in **C)** NaPhos and **D)** 100% ammonium sulfate for native Lyz (black circle), Lyz(3+) (gray half open circle), DP 20 (red half open square), and DP 66 (blue half open square). Conjugates with 1 initiator in **E)** NaPhos and **F)** 100% ammonium sulfate for native Lyz (black circle), Lyz(1+) (gray open circle), DP 14 (red open square), and DP 44 (blue open square). Blanks from auto hydrolysis of substrate are shown in NaPhos and 100% ammonium sulfate (brown circles) in plots **A)** and **B)**, respectively.

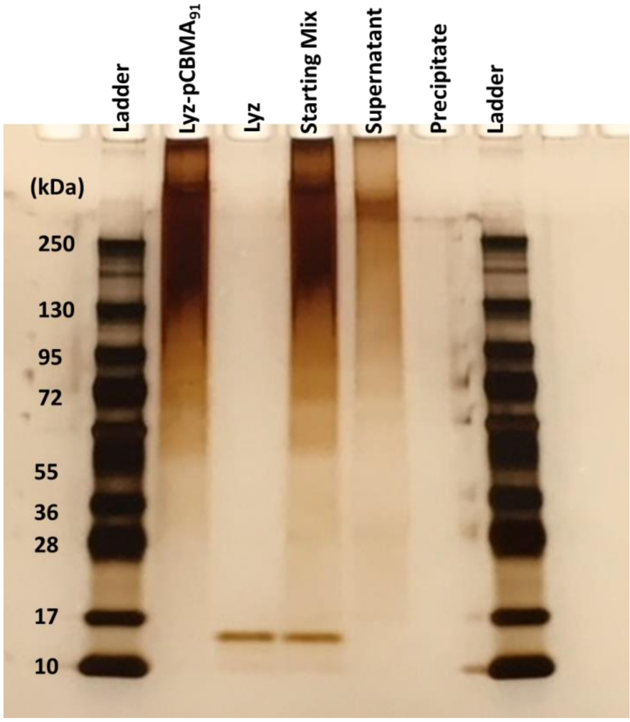

Supplementary Figure 15. Silver stained SDS-PAGE analysis from a second round of purification of Lyz(5+)pCBMA DP 91 from a mixture with native Lyz. The supernatant from Figure 6A was purified again by the addition of 100% saturated ammonium sulfate and the same processing was performed as in Figure 6A. No native Lyz remained in the supernatant after the 2nd purification.

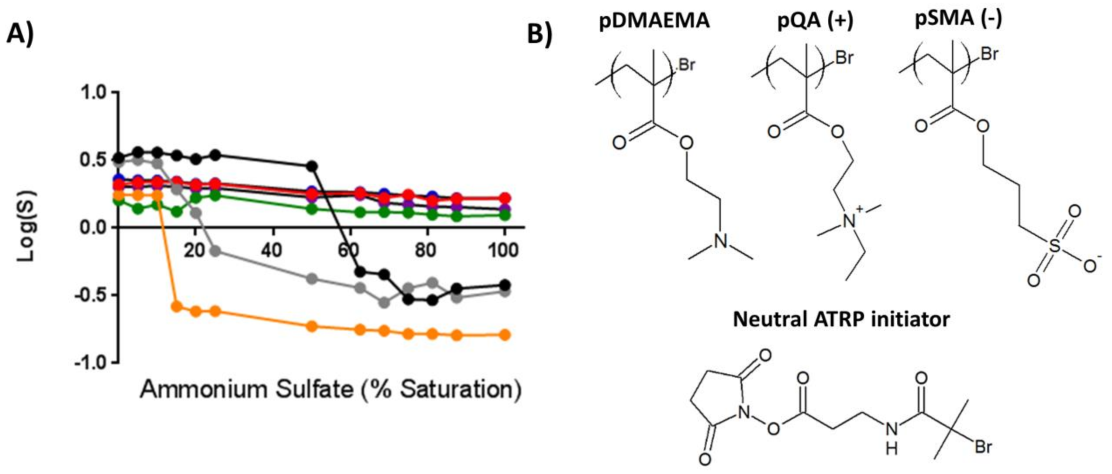

Supplementary Figure 16. A) Ammonium sulfate precipitation of native CT, CT-neutral initiator, and CT-polymer conjugates. Native CT (black circle), CT-neutral initiator (gray circle), CT-pCBMA DP 112 (red circle), CT-pOEGMA DP 97 (orange circle), CT-pDMAEMA DP 89 (green circle), CT-pQA DP 89 (blue circle), and CT-pSMA DP 113 (purple circle). **B)** The various structures of charged polymers that were grown from CT using the neutral ATRP initiator. Error bars (within the symbols) represent the standard deviations from triplicate measurements.

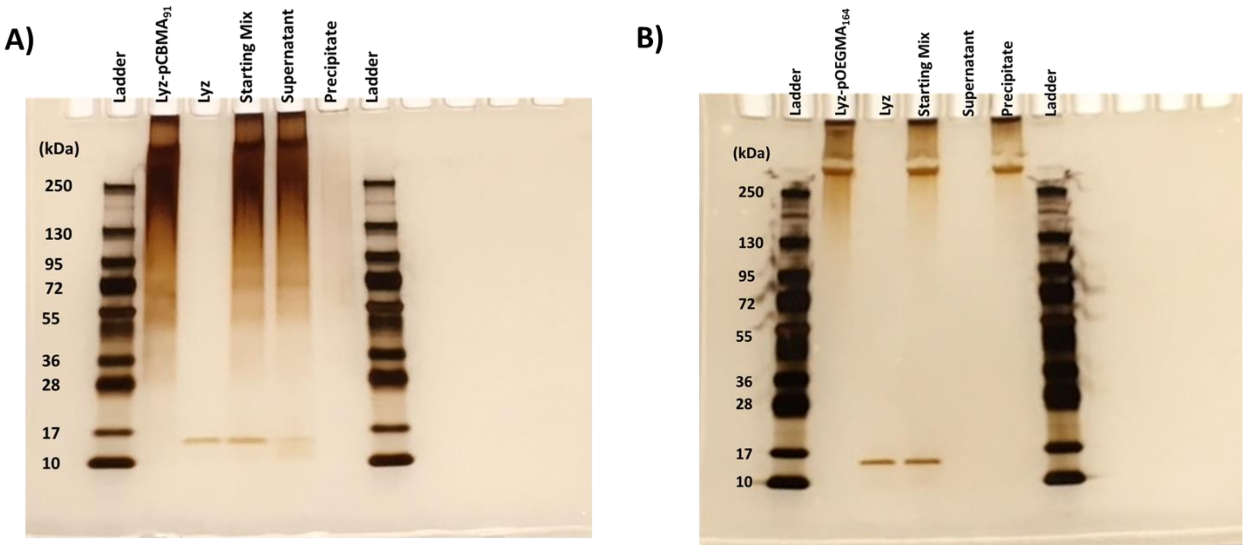

Supplementary Figure 17. Uncropped and unprocessed SDS-PAGE gels from Figure 5 in the main text.

Supplementary Tables

Supplementary Table 1. Lyz-polymer characterization table for conjugates with 1 initiator (1+) or 3 initiators (3+).

	**Estimated DP***	**D_h (nm; number dist.)**
**Lyz(1+)**	--	3.3 ± 0.3
**Lyz(3+)**	--	3.7 ± 0.2
**Lyz(1+) pCBMA**	14	5.2 ± 0.8
	44	6.0 ± 0.8
**Lyz(1+) pOEGMA**	9	5.9 ± 0.8
	93	14.0 ± 2.5
**Lyz(3+) pCBMA**	20	5.3 ± 1.1
	66	12.7 ± 1.5
**Lyz(3+) pOEGMA**	16	7.9 ± 1.5
	57	18.0 ± 3.3

*DP was estimated from BCA results.

Supplementary Table 2. Enzymatic activities of Lyz, Lyz-initiators, and Lyz-pCBMA conjugates of increasing DP in 50 mM NaPhos buffer and 100% saturated ammonium sulfate (4.1 M). Activity was measured using the fluorescent substrate 4-Methylumbelliferyl β-D-N,N′,N″-triacetylchitotrioside over 4 h. Data were fit to linear regressions to obtain the reaction rate. Error represents the standard deviations from triplicate measurements.

		Reaction Rate (RFU min^{-1})		
		**50 mM NaPhos (pH 6.0)**	**4.1 M Ammonium Sulfate (pH 5.5)**	**Ratio (Ammonium Sulfate: NaPhos)**
	**Lyz**	26.5 ± 1.5	25.6 ± 1.5	1.0
5 initiators	**Lyz(5+)**	18.8 ± 3.8	78.4 ± 4.3	4.2
	**DP 18**	19.3 ± 1.3	30.7 ± 2.0	1.6
	**DP 32**	33.9 ± 4.4	36.3 ± 1.6	1.1
	**DP 56**	21.3 ± 2.6	26.6 ± 1.1	1.2
	**DP 79**	21.3 ± 0.2	28.2 ± 0.9	1.3
	**DP 91**	17.9 ± 2.2	24.2 ± 0.7	1.4
3 initiators	**Lyz(3+)**	30.6 ± 0.6	74.1 ± 1.5	2.4
	**DP 20**	35.8 ± 0.3	73.2 ± 2.3	2.0
	**DP 66**	19.0 ± 0.1	30.1 ± 0.6	1.6
1 initiator	**Lyz(1+)**	30.1 ± 0.1	53.4 ± 1.3	1.8
	**DP 14**	43.7 ± 0.3	78.6 ± 1.4	1.8
	**DP 44**	28.3 ± 1.2	48.5 ± 0.8	1.7
