# Supplementary material for: Transforming protein-polymer conjugate purification by tuning protein solubility
Source: Nat Commun. 2019 Oct 17;10:4718. doi: 10.1038/s41467-019-12612-9 (PMC6797786; doi:10.1038/s41467-019-12612-9)
Supplement: Supplementary file 2 — Description of Additional Supplementary Files [file 41467_2019_12612_MOESM2_ESM.pdf]

Description of Additional Supplementary Files

File Name: Supplementary Movie 1
Description: Lyz(5+)pOEGMA DP 25 showing polymer conformations in 0.0 M NaCl (grey polymer chains) overlapped with polymer conformations in 5.0 M NaCl (blue polymer chains). Polymer chains collapse onto the protein surface with increasing salt concentration.
